# High Content Phenotypic Cell-Based Visual Screen Identifies *Mycobacterium tuberculosis* Acyltrehalose-Containing Glycolipids Involved in Phagosome Remodeling

**DOI:** 10.1371/journal.ppat.1001100

**Published:** 2010-09-09

**Authors:** Priscille Brodin, Yannick Poquet, Florence Levillain, Isabelle Peguillet, Gerald Larrouy-Maumus, Martine Gilleron, Fanny Ewann, Thierry Christophe, Denis Fenistein, Jichan Jang, Mi-Seon Jang, Sei-Jin Park, Jean Rauzier, Jean-Philippe Carralot, Rachel Shrimpton, Auguste Genovesio, Jesus A. Gonzalo-Asensio, Germain Puzo, Carlos Martin, Roland Brosch, Graham R. Stewart, Brigitte Gicquel, Olivier Neyrolles

**Affiliations:** 1 Biology of Intracellular Pathogens Inserm Avenir Group, Institut Pasteur Korea, Seongbuk-gu, Seoul, Korea; 2 UP Integrated Mycobacterial Pathogenomics, Institut Pasteur, Paris, France; 3 Centre National de la Recherche Scientifique, Institut de Pharmacologie et de Biologie Structurale, Toulouse, France; 4 Université de Toulouse, Université Paul Sabatier, Institut de Pharmacologie et de Biologie Structurale, Toulouse, France; 5 Screening Technologies and Pharmacology, Institut Pasteur Korea, Seongbuk-gu, Seoul, Korea; 6 Image Mining, Institut Pasteur Korea, Seongbuk-gu, Seoul, Korea; 7 Unit of Mycobacterial Genetics, Institut Pasteur, Paris, France; 8 Division of Microbial Science, Faculty of Health and Medical Sciences, University of Surrey, Guildford, Surrey, United Kingdom; 9 Department of Microbiology and Public Health, Faculty of Medicine, University of Zaragoza, Zaragoza, Spain; University of New Mexico, United States of America

## Abstract

The ability of the tubercle bacillus to arrest phagosome maturation is considered one major mechanism that allows its survival within host macrophages. To identify mycobacterial genes involved in this process, we developed a high throughput phenotypic cell-based assay enabling individual sub-cellular analysis of over 11,000 *Mycobacterium tuberculosis* mutants. This very stringent assay makes use of fluorescent staining for intracellular acidic compartments, and automated confocal microscopy to quantitatively determine the intracellular localization of *M. tuberculosis*. We characterised the ten mutants that traffic most frequently into acidified compartments early after phagocytosis, suggesting that they had lost their ability to arrest phagosomal maturation. Molecular analysis of these mutants revealed mainly disruptions in genes involved in cell envelope biogenesis (*fadD28*), the ESX-1 secretion system (*espL*/Rv3880), molybdopterin biosynthesis (*moaC1* and *moaD1*), as well as in genes from a novel locus, Rv1503c-Rv1506c. Most interestingly, the mutants in Rv1503c and Rv1506c were perturbed in the biosynthesis of acyltrehalose-containing glycolipids. Our results suggest that such glycolipids indeed play a critical role in the early intracellular fate of the tubercle bacillus. The unbiased approach developed here can be easily adapted for functional genomics study of intracellular pathogens, together with focused discovery of new anti-microbials.

## Author Summary

One of the major virulence mechanisms of the tuberculosis bacillus, *Mycobacterium tuberculosis*, is its ability to resist killing by phagocytic cells of the host immune system, namely the macrophages. Macrophages degrade invading microbes by engulfment inside a vacuole, or phagosome, that progressively acidifies and accumulates hydrolytic properties. *M. tuberculosis* has the unique ability to block phagosome maturation and acidification. To identify mycobacterial genes involved in phagosome maturation arrest, we developed a novel high-throughput technology based on automated confocal microscopy. We screened a library containing over 11,000 *M. tuberculosis* mutants, and we could identify 10 mutants that had lost their ability to resist phagosome acidification. Genetic characterization of these mutants revealed that they carried lesions in genes involved in various cell processes, including biogenesis of the cell envelope. In particular, two independent mutants in the same genetic locus showed altered production of two lipids, namely diacyltrehalose (DAT) and sulfoglycolipid (SGL). *In vitro* experiments showed that SGL can indeed influence phagosome maturation. Our study unravels the role of novel lipid molecules in mycobacterial intracellular parasitism; our approach may be useful to identify virulence genes in other intracellular pathogens, and to identify novel antimicrobials.

## Introduction

Upon engulfment by host macrophages, *M. tuberculosis*, the main etiological agent of tuberculosis in humans, localizes in vacuoles or phagosomes that fail to fuse with host cell lysosomes and to acidify [1], [2]. This process, called phagosome maturation arrest, may contribute to mycobacterial pathogenicity and to mycobacterial evasion from host immune surveillance. Molecular determinants for the specific features of this host-pathogen interaction are starting to be elucidated, though no clear picture has yet been established [1], [2], [3], [4], [5]. Several mycobacterial products affecting phagosome maturation, such as cell wall lipoglycans and glycolipids [6], or allowing resistance to the acidic milieu of the phagosome [7] have been reported. Apart from focused, candidate gene-based studies [8], [9], large scale approaches have been performed using mutant libraries to obtain a comprehensive picture of the process. Pethe *et al.* used a *M. tuberculosis* CDC1551 transposon mutant library to identify mutants that fail to prevent phagosome-lysosome fusion [10]. With the same aim, by use of flow cytometry, Stewart *et al.* identified a series of *Mycobacterium bovis* BCG mutants that fail to prevent phagosome acidification [11]. Both studies generated comprehensive lists of novel mycobacterial genes possibly involved in phagosome maturation arrest. However they were performed by infecting cells with large pools of mutants and necessitated several rounds of amplification, thereby introducing a mutant selection bias. Indeed mutants that traffic into late endosomal compartments are likely to be impaired in growth and could be lost during the amplification process. Moreover, such competitive infections may miss detection of mutants that can be trans-complemented by other clones within the mixed infection. This may occur when the interrupted gene encodes a secreted virulence factor or a factor that interferes with processes involving secretion of host factors.

This led us to develop a new type of screening system whereby mutants would be individually investigated, in the absence of other competitive strains. We took advantage of automated confocal fluorescence microscopy and dedicated image analysis to monitor sub-cellular mycobacterial localization of a large number of samples. A transposon mutant library made in a virulent clinical isolate of *M. tuberculosis* of the W/Beijing family and containing over 11,000 individual mutants was used to infect macrophages *in vitro*. Ten mutants with an impaired ability to prevent phagosome acidification were isolated. Strikingly, out of these ten mutants, two carried independent insertions in the adjacent *moaC1* and *moaD1* genes, and two further mutants carried independent insertions in Rv1503c and Rv1506c located within the so-called *losA* locus that is involved in the synthesis of lipooligosaccharides (LOS) in another mycobacterial species, *Mycobacterium marinum* [12], [13]. This prompted us to further investigate the lipid content of the concerned transposon mutants, which allowed us to identify novel lipids that are involved in the acidification of phagosomes. The results of this study thus strenghten the hypothesis that *M. tuberculosis* modulate the biosynthesis of specific glycolipids to manipulate phagosome maturation and shed new light on the genetic locus and the synthesis pathways involved.

## Results

### Positive LysoTracker staining of macrophages correlates with presence of *M. tuberculosis* in acidified phagosomes

**Figure 1:**
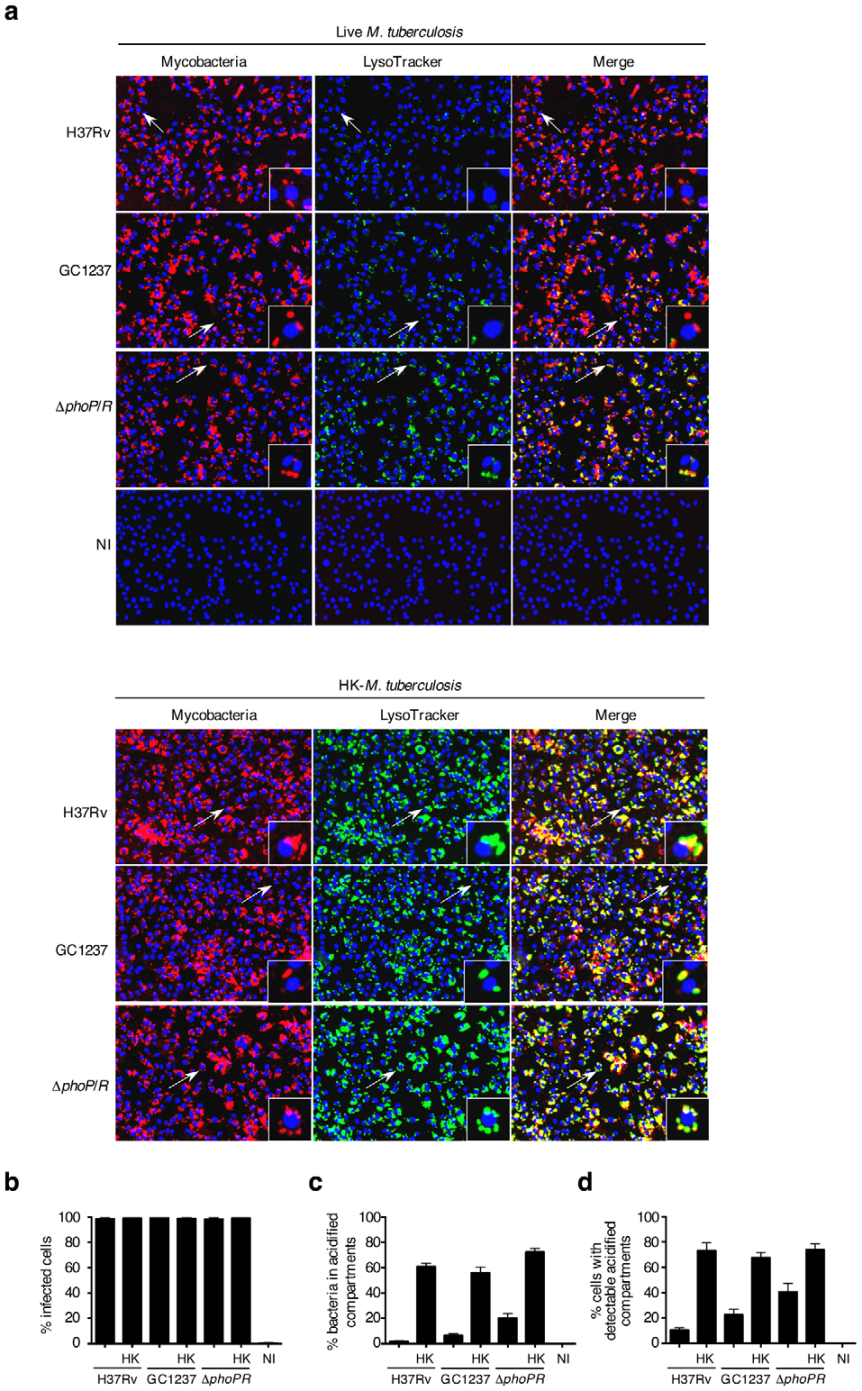
Quantification of *M. tuberculosis* intracellular trafficking into acidified compartments by automated confocal imaging. (**a**) Representative pictures of mouse bone-marrow-derived macrophages infected with CypHer5 (red)-labeled *M. tuberculosis* H37Rv, GC1237, or GC1237-derived Δ*phoP/R* mutant, or heat-killed (HK) bacteria. After 2 hours of infection, cells were stained with LysoTracker DND-26 (green) and DAPI (blue) for acidic compartments and cell nuclei labeling, respectively. Non-infected macrophages (NI) are shown as control. Images span 0.450×0.340 mm^2^. Arrows indicate cells shown at higher magnification in the insets. (**b-d**) Percentage of infected cells, bacteria inside LysoTracker-positive acidified compartments, and cells with LysoTracker-positive compartments. Values for each condition are the average ± s.d. determined for seven different infections for which four fields were recorded and analyzed, and are representative of two independent experiments.

**Figure 2:**
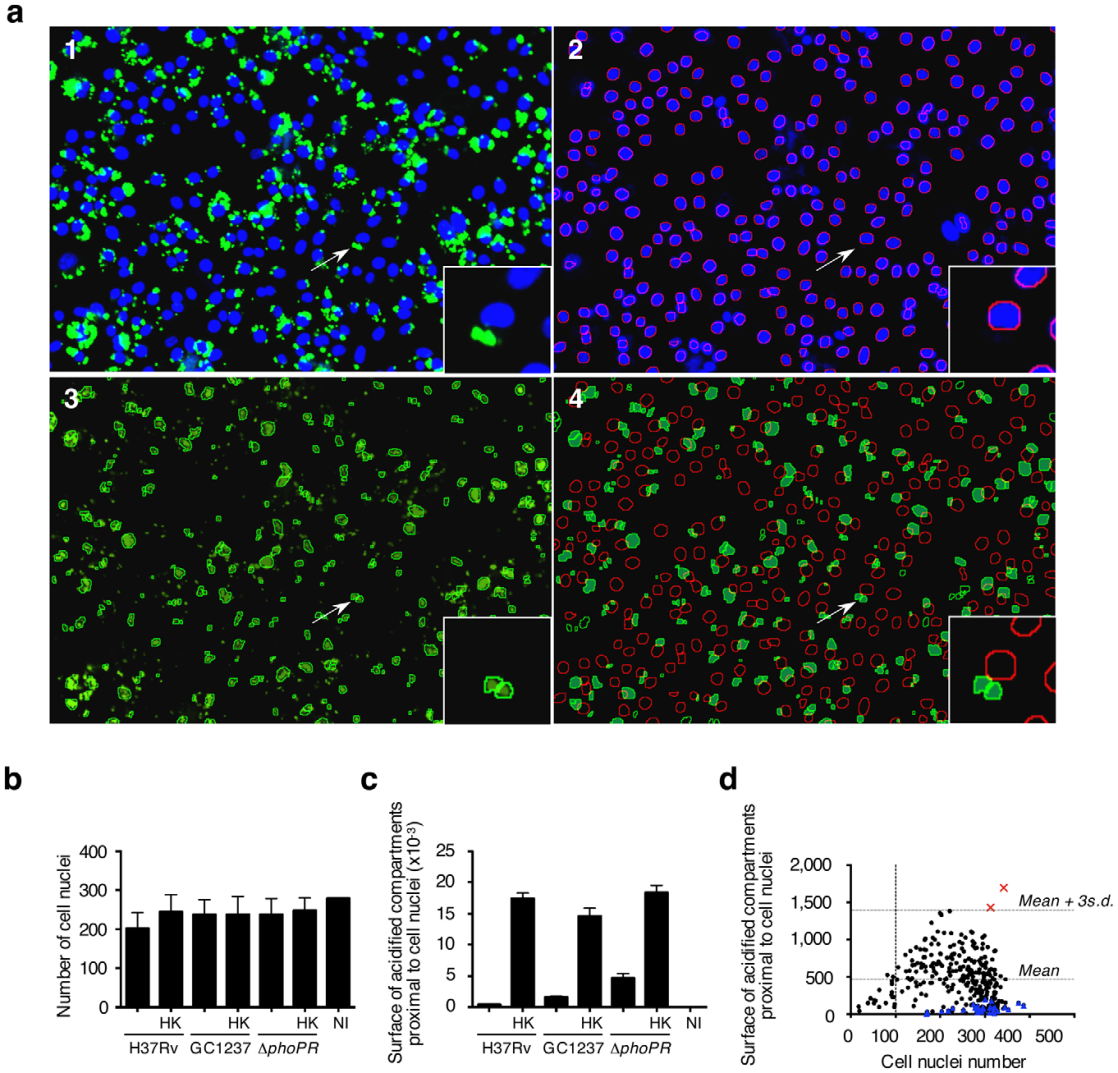
Phenotypic cell-based genetic screen. (**a**) Mouse bone marrow-derived macrophages were infected with HK-*M. tuberculosis* H3Rv. Image analysis. **1**: Typical 2-color image of LysoTracker (green)- and DAPI (blue)-stained infected cells; 2: Red-circled blue objects correspond to detected cell nuclei from the blue channel image; 3: Green-circled green objects correspond to detected acidic compartments from the green channel image; 4: Green-filled surfaces correspond to acidic compartments detected as being proximal to red-circled cell nuclei. Images span 0.450×0.340 mm^2^. (**b,c**) Image-based quantification of the cell nuclei number and the total acidic compartment surface proximal to cell nuclei. Values for each condition are the average ± s.d. determined for seven different infections for which four fields were recorded and analyzed, and are representative of three independent experiments and of controls from each 384-well screening microplates. Abbreviations are as in Figure 1. (**d**) Library screening. Automated confocal imaging-based analysis of a selected microplate containing mouse bone marrow-derived macrophages infected for 2 h with a subset of the *M. tuberculosis* GC1237-derived mutant library (black dots & red squares), or left uninfected (blue triangles), and subsequently labeled with LysoTracker. Red crosses correspond to two hit mutants selected from this particular microplate.

In order to set-up the optimal conditions of *M. tuberculosis* infection, mouse bone marrow-derived macrophages were infected with mycobacteria that had previously been covalently labeled with the red fluorescent dye CypHer5. *M. tuberculosis* reference strain H37Rv, the W-Beijing strain GC1237 [14] and a GC1237 Δ*phoP/R* attenuated mutant [15] as well as heat-killed bacteria were used. After 2 h of infection, macrophages were pulsed with the acidotropic green fluorescent dye LysoTracker DND-26 to label the acidified compartments. After fixation and nuclei labeling, sample images from four fields per well were acquired using an automated confocal microscope. For each field, three images were recorded: one for the cell nuclei (blue-channel), one for the CypHer5 labeled mycobacterium-positive compartment (red-channel) and one for the LysoTracker-DND-26 positive compartment (green-channel). The acquisition parameters were set so that the LysoTracker signal was minimal in resting non-infected cells. In these settings, cells infected with heat-killed bacteria exhibited a strong LysoTracker signal, whereas cells infected with live bacilli showed a weak signal (Figure 1a). This first set of experiments led us to investigate whether the sole LysoTracker signal could be used as a positive indirect readout for the presence of mycobacteria in acidified compartments. An image analysis script was then developed to determine the parameters that best describe the images and that would be a correlate of infected cells, bacterial load, and LysoTracker-positive compartments. First, the nuclear stain DAPI that was used enabled accurate macrophages number quantification and their spatial positioning. Next, spots in either the red-channel or the green-channel images were labeled as bacterium- or LysoTracker-positive objects, respectively, if their size surface was at least 3 pixels with intensity above a defined threshold. Because the nuclear labeling of the macrophage did not allow us to delineate the exact contour of the cell, a macrophage was considered infected if labeled bacterium objects were distant of less than 5 pixels from the DAPI signal surface. Similarly, LysoTracker-positive objects were labeled as acidified compartments if they were found distant of less than 5 pixels from the cell nucleus in our acquisition conditions. Nearly 100% of the cells were found to be infected after 2 h (Figure 1b). The fraction of bacteria co-localizing with LysoTracker-positive staining (Figure 1c) was found to be less than 10% in cells infected with *M. tuberculosis* H37Rv (2±1%) or GC1237 (7±1%). In contrast, upon infection with heat-killed H37Rv or GC1237, more than 50% of the bacteria were found to co-localize with acidified compartments (61±3% and 56±4%, respectively). In addition, 20±3% of the live attenuated Δ*phoP/R* mutant showed a marked acidification of their vacuoles. Opposite patterns were observed when infected cells were stained for the early endosomal marker Rab5A (Figure S1a). Of particular interest, quantification of the percentage of cells containing detectable acidified compartments led to similar results between strains and conditions (Figure 1d). In addition to this latter, another parameter deduced from the images is the surface of acidified compartments proximal to cell nuclei that refers to the total surface area of all the Lysotracker positive objects that are distant of less than 5 pixels from the DAPI signal surface in each field (Figure 2a).

The dynamic range of the assay was confirmed by monitoring the dose-response of Zymosan A, a substance that is known to rapidly traffic into lysosomes. With increasing concentrations of Zymosan A, there was a subsequent linear increase in the intracellular surface positive for LysoTracker staining (Figure S1b,c). This further demonstrates that the acidified compartment surface parameter is directly proportional to the amount of particles within acidic vacuoles and can be used as a correlate of the localization of mycobacteria in acidified vacuoles without additional staining and detection of the bacilli.

To simplify the protocol for high throughput screening (HTS), macrophages were distributed into 384-well plates that had previously been inoculated with mycobacteria. Infection was carried out at 37°C for 2 h, a time-lapse sufficient for macrophage adherence. The monolayer was then subjected to LysoTracker and nuclei staining as described above. The day-to-day as well as plate-to-plate reproducibility was ascertained by including live and heat-killed *M. tuberculosis* H37Rv, GC1237 and Δ*phoP/R* mutant in each plate. All samples corresponding to the various reference strains contained a similar number of cell nuclei (Figure 2b). The surface of acidified compartments proximal to cell nuclei in live samples was significantly lower than in heat-killed samples with a Z' score value (live *vs*. heat-killed) above 0.2 (Figure 2c). Thus this parameter was further chosen as a read-out for HTS.

Our screening assay therefore consisted in the search for bacterial mutants that induce a positive LysoTracker staining phenotype upon macrophage infection.

### Identification of transposon mutants that fail to induce phagosome maturation arrest

An 11,180-member mutant library was constructed by transposition of the IS*1096*-derived Tn*5367* transposon in the *M. tuberculosis* W-Beijing strain GC1237 as previously described [16]. Transposon site hybridization (TraSH) analysis of a 500-member pool subset of the library showed that insertions occurred uniformly in the chromosome (Figure S2). Given that the *M. tuberculosis* genome spans around 4,000 open reading frame [17], our library represents a 3X-coverage of the whole genome. The mutant library was formated into fourty-four 384-well microplates, which included 5 control wells for each of *M. tuberculosis* GC1237, the Δ*phoP/R* mutant, or heat killed GC1237. In addition, all plates included a set of wells with non-infected cells.

**Figure 3:**
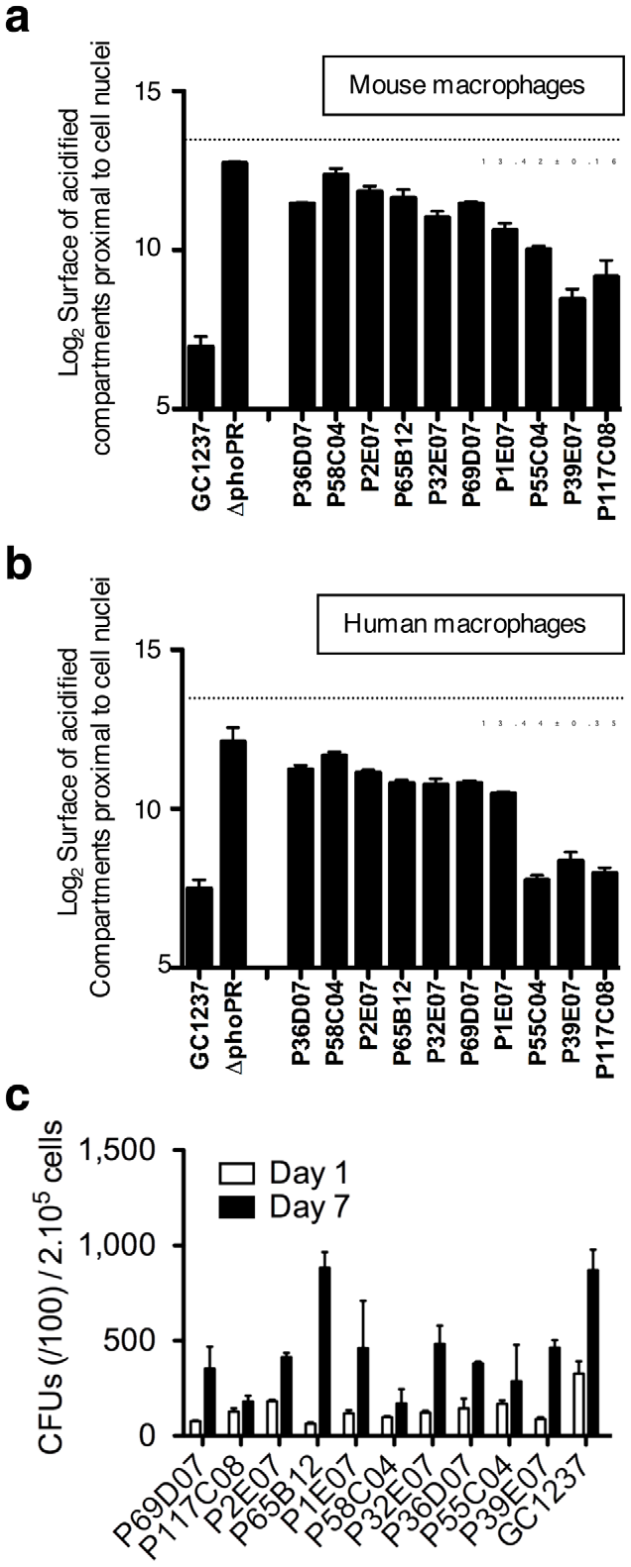
Phenotypic cell-based assay identifies 10 *M. tuberculosis* mutants that rapidly traffic into acidified phagosomes. Mouse bone marrow- (**a**) and human monocyte- (**b**) derived macrophages were infected for 2 h with the various mutants at a multiplicity of infection (MOI) of 10 bacteria per cell, stained with LysoTracker and subsequently analyzed by confocal imaging. Cells infected with *M. tuberculosis* GC1237, heat-killed (HK)-GC1237 or with the Δ*phoPR* mutant are shown as controls. Values for each condition are the average ± s.d. determined for three different infections for which four fields were recorded and analyzed, and are representative of two independent experiments. The dotted lines and the numbers below the lines indicate values for HK-bacteria. (c) Intracellular survival ability of the mutants. Mouse bone marrow-derived macrophages were infected with the mutant and wild type strains at a MOI of 1 bacterium per cell. CFUs were scored 1 and 7 days after infection. The bars for each condition are the mean ± s.d of a triplicate experiment.

The validation of the screen (Z' score value for live *vs*. heat-killed >0.2) was first ascertained by control analysis on each microplate. Mutants were selected using a stringent threshold, *i.e.* for values of acidified compartment surface of 3 s.d. above the average, and for nuclei numbers above 100, which corresponds to low cytotoxicity. A typical plate is shown in Figure 2d, whereby two mutants with aberrant trafficking are highlighted. From this analysis, a series of 10 mutants was selected for further characterization. The trafficking phenotype of all selected mutants was confirmed on the individual level in mouse and human primary macrophages (Figure 3). The ability of the ten mutants to survive and replicate inside mouse macrophages was investigated to determine whether enhanced trafficking into acidified compartments correlated with impaired intracellular growth (Figure 3c). Strikingly, intracellular trafficking to acidified vacuoles did not necessarily correlate with growth attenuation; indeed the mutant in *lppM* (mutant P65B12) for instance, that largely trafficked to an acidified vacuole, could actively multiply inside host cells on the longer term.

**Figure 4:**
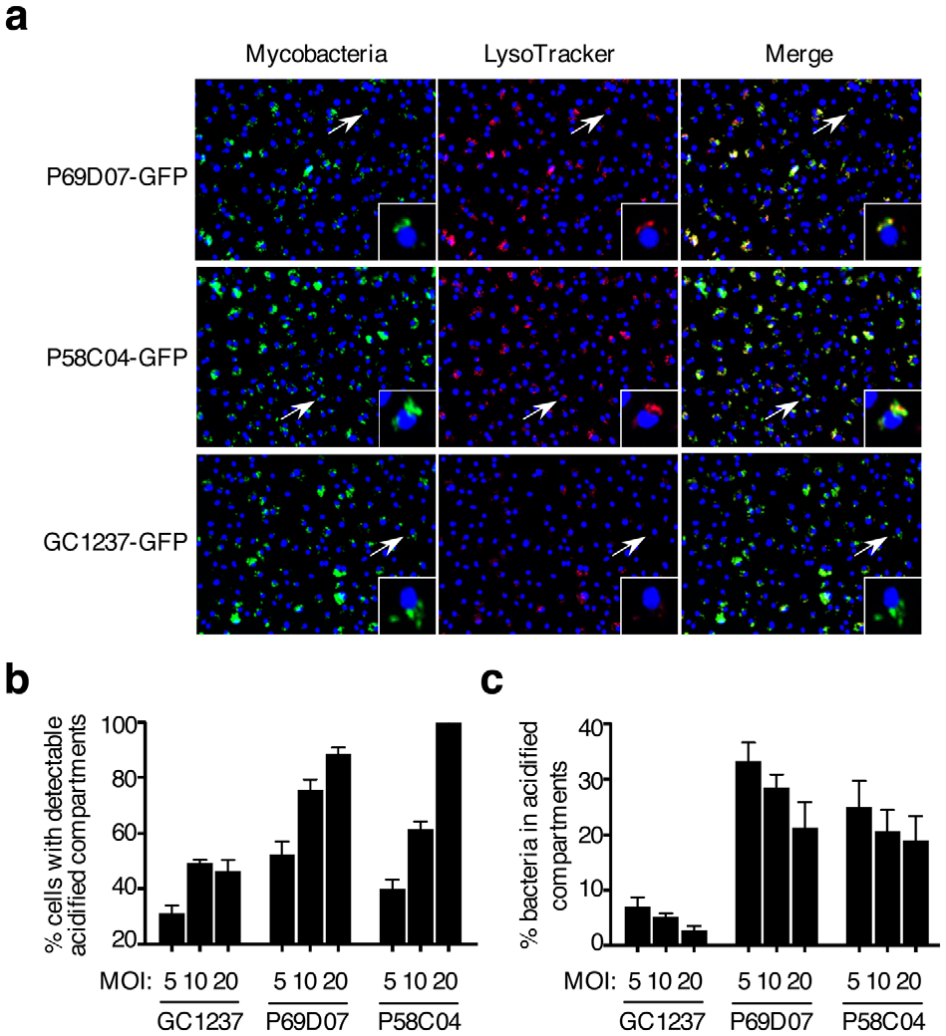
*M. tuberculosis* mutants #1 and #6 traffic rapidly to acidified vacuoles. (**a**) Confocal microscopy analysis of LysoTracker (red)-stained mouse bone marrow-derived macrophages infected for 2 h with GFP-expressing *M. tuberculosis* #1 and #6 mutants and GC1237. Images span 0.450×0.340 mm^2^. (**b,c**) Image-based quantification of bacteria inside LysoTracker-positive acidic compartments, and cells with LysoTracker-positive acidic compartments for different multiplicities of infection (MOI). Values for each condition are the average ± s.d. determined for three different infections for which four fields were recorded and analyzed, and are representative of two independent experiments.

As selected examples of individual confirmation of altered trafficking, two mutants are displayed in Figure 4, where mouse macrophages were infected with GFP-expressing strains, subsequently stained with LysoTracker red, and examined by confocal microscopy (Figure 4a). Image analysis confirmed that the percentages of acidified compartment-containing cells were higher for the selected mutants than for the GC1237 parental wild-type strain (Figure 4b), which correlated with higher percentages of bacteria in acidic vacuoles (Figure 4c). Indeed more than 25% of the mutant bacilli were found in acidic vacuoles, compared to less than 10% for the control strain.

Genes disrupted by transposon insertion in the 10 selected mutants were identified by ligation-mediated PCR and sequencing (Table 1) [16]. Among others, genes involved in cell envelope biogenesis (*fadD28*, *ppe54*, *lppM*), phosphate uptake (*pstS3*), ESX-1-mediated secretion (*espL*/Rv3880) and molybdopterin biosynthesis (*moaC1*/*moaD1*) were found. The maturation stage of the mycobacterial phagosome was further characterized for two mutants (in Rv1503c and Rv1506c) by immunofluorescence and confocal microscopy observation. Similarly to the phagosomes of wild type *M. tuberculosis*, the phagosomes of mutant strains were mostly negative for the lysosomal marker CD63 (Figure S3a). The fraction of v-ATPase-positive (Figure S3b) and LAMP-1-positive (data not shown) phagosomes was increased in cells infected with the mutants, as compared to the wild-type strain, which is in agreement with our results using LysoTracker. Genetic complementation of the ten mutants was achieved using pYUB412-derived integrating cosmids [18]. The different cosmids carry large size DNA fragments from *M. tuberculosis* Erdman, which encompass the corresponding region in the genome of *M. tuberculosis* H37Rv (Table 2). Trafficking of all the complemented mutant strains was either fully or at least partially restored, as assessed by LysoTracker staining of infected macrophages (Table 1). Our results confirm that phagosome maturation arrest and intracellular survival are multifactorial processes involving a combination of mycobacterial genes [9], [10]. In addition, we show that increased phagosome maturation does not necessarily correlate with mycobacterial stasis or killing in the infected cells. As none of the 10 trafficking mutants showed a growth defect in culture broth (Figure S4), these processes seem to have evolved during adaptation of tubercle bacilli to the mammalian host.

**Table 1:** List of confirmed mutants that rapidly traffic into acidified compartments.

Mutant id	Gene (nt^a^)	Putative function	Trafficking into acidified phagosomes^b^	
			Tn mutant	Complemented
P69D07	*pstS3* (482)	Phosphate transport	72±4	29±2
P117C08	Rv1503c (186)	TDP-4-oxo-6-deoxy-D-glucose transaminase (glycosyl aminotransferase)	35±5	3±0
P2E07	Rv1506c (211)	Methyltransferase	83±3	32±1
P65B12	*lppM* (272)	Lipoprotein of unknown function	83±5	33±2
P1E07	Rv2295 (-35)	Unknown	62±4	7±0.5
P58C04	*fadD28* (1252)	DIM biosynthesis	91±3	0±0
P32E07	*moaC1* (507)	Molybdopterin biosynthesis	69±2	0±0
P36D07	*moaD1* (49)	Molybdopterin biosynthesis	72±2	0±0
P55C04	*ppe54* (1126)	Unknown	55±6	0±0
P39E07	Rv3880c (269)	Unknown	29±10	5±2

a nt indicates the position of Tn insertion in nucleotides.

b indicates the mean % ± s.d. of trafficking into acidified vacuoles relative to *M. tuberculosis* Heat killed control (100%) based on surface of acidic compartments proximal to cell nuclei data shown in Figure 3a. Results are the average (+/- s.d.) of at least three independent experiments.

The finding that two genomic loci (*moaC1*/*D1 and* Rv1503/06c) were each represented by two independent transposon mutants suggests that the identified regions indeed play key roles in the phagosome maturation arrest induced by *M. tuberculosis*. These results highlight the power and stringency of our screening method and prompted us to investigate one of the identified loci (Rv1503c/06c) in more detail. Indeed this genomic region is similar to a locus in the closely related fish pathogen *Mycobacterium marinum* that is encoding enzymes involved in LOS synthesis [12], [13], which might play important roles in host-pathogen interaction.

### Acyltrehalose-containing glycolipids are involved in early phagosome maturation arrest

**Figure 5:**
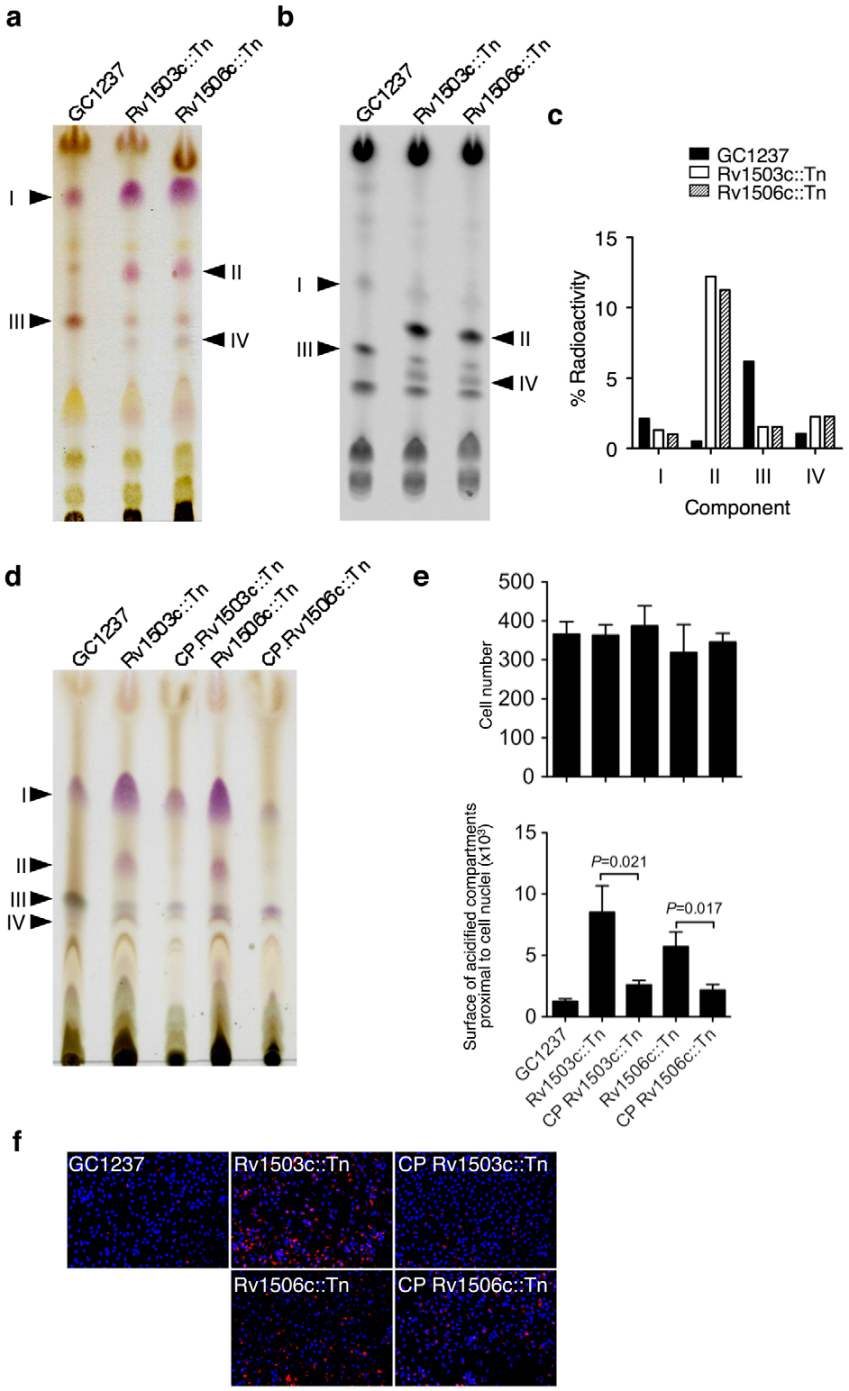
Lipid content and phenotype of the *M. tuberculosis* GC1237 wild-type, Rv1503c::Tn and Rv1506c::Tn mutants, and complemented strains. (**a,b**) anthrone-stained (a) and autoradiogram (b) of thin-layer chromatography of [1-^14^C]propionate labelled lipids (20000 cpm/lane). (**c**) Quantification of the lipids from (b). One representative out of four experiments is shown. (**d**) TLC analysis of the lipids of cosmid-complemented strains *vs* wild-type and mutant strains. (**e,f**) Trafficking phenotype of the wild-type, mutant and complemented strains. Representative pictures of mouse bone-marrow-derived macrophages infected with *M. tuberculosis* GC1237, Rv1503c::Tn and Rv1506c::Tn mutants, and complemented strains. After 2 hours of infection, cells were stained with LysoTracker DND-99 (red) and DAPI (blue) for acidic compartments and cell nuclei labeling, respectively. In (a), lipids were subjected to TLC with CHCl_3_/CH_3_OH/H_2_O 30/8/1 (v/v/v) as the solvent; in (b) and (d), lipids were subjected to TLC with CHCl_3_/CH_3_OH/H_2_O 35/8/1 (v/v/v) as the solvent.

**Figure 6:**
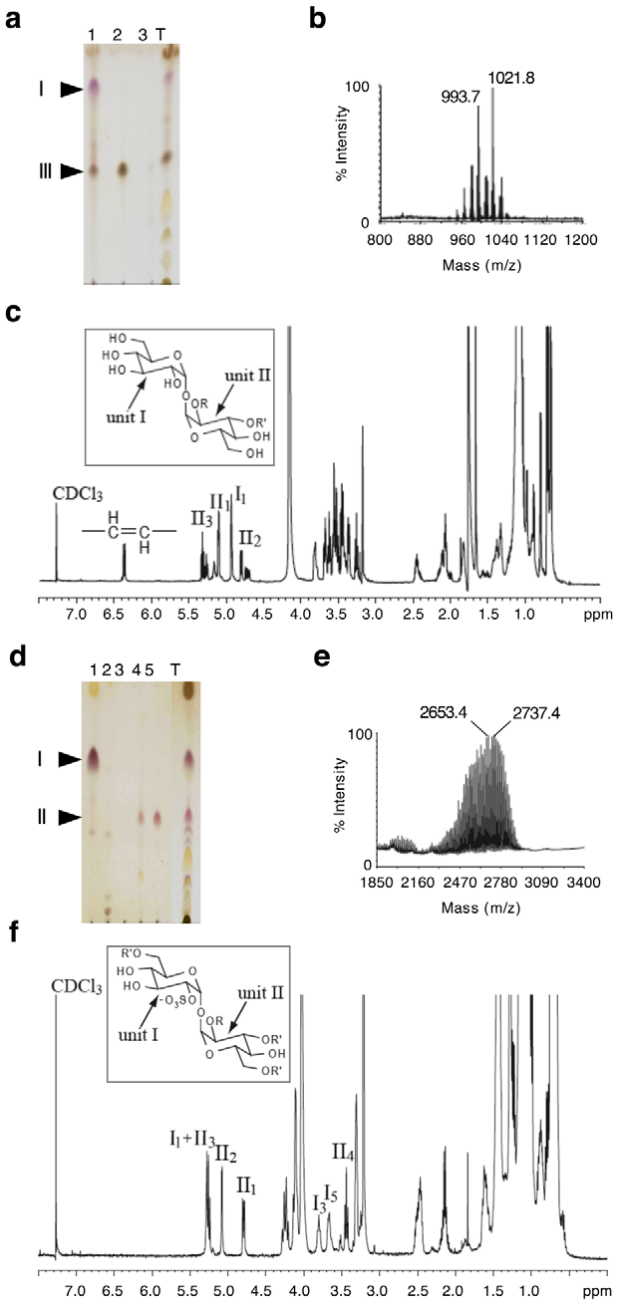
Identification of lipids II and III as Ac_4_SGL and DAT respectively. (**a,d**) TLC analysis of fractions from an anion exchange column loaded with total chloroform extract (T) eluted with CHCl_3_ (1), CHCl_3_/CH_3_OH 9/1 (2), CHCl_3_/CH_3_OH/H_2_O 60/35/8 (3), chloroform/methanol 1/2 containing 0.1 M ammonium acetate (4), chloroform/methanol 1/2 containing 0.3 M ammonium acetate (5). In (a), lipids were extracted from the GC1237 strain. In (d), lipids were extracted from the Rv1506c mutant strain. (**b,e**) MALDI-MS spectra of DAT and Ac_4_SGL, respectively. The spectrum of DAT was recorded in positive mode while the spectrum of Ac_4_SGL was recorded in negative mode. (**c,f**) 1D ^1^H NMR spectra (∂ ^1^H: 0-7.5) of DAT and Ac_4_SGL, respectively. I_1_ stands for proton 1 of unit I. The characteristic protons are interpreted on the figure. Insets in (c) and (f) show the structure of DAT and Ac_4_SGL, respectively. R corresponds to palmitic or stearic acids while R' corresponds to polymethyl-branched fatty acid. The two glucose units are labeled I and II.

We further characterized the mutants in Rv1503c and Rv1506c. These genes encode a putative TDP-4-oxo-6-deoxy-D-glucose transaminase and a putative methyltransferase, respectively, as evaluated by comparative bioinformatic analyses. They are located in a locus that resembles the lipooligosaccharides (LOS) locus of *M. marinum* [12], [13], which is partially conserved between the two species. Interestingly, the Rv1503c-to-Rv1507c genes belong to a single operon, as assessed by RT-PCR amplification of the gene junctions (data not shown). Rv1503c and Rv1506c share 89% amino acid similarity with their *M. marinum* orthologs MMAR2320/*wecE* and MMAR2322, respectively. We first hypothesized that this locus may be involved in the synthesis of LOS or LOS-related glycolipids in *M. tuberculosis*. However with the exception of particular strains of the *M. canetti* family [19], [20] which represents rare and more distantly related tubercle bacilli [21], LOS have not been identified in tubercle bacilli. In agreement with these data, we did not detect any LOS production in *M. tuberculosis* GC1237 (data not shown). Thus, we next hypothesized that the gene cluster under study may be involved in the synthesis of other forms of acyltrehaloses. Lipid extracts from bacterial cells were examined by TLC upon anthrone staining (Figure 5a) or metabolic labeling of methyl-branched fatty acid-containing lipids with [1-^14^C]propionate (Figure 5b,c). These analyses revealed that the Rv1503c::Tn and Rv1506c::Tn mutants markedly overproduced a molecule (named “II” in Figure 5a-d), whereas they produce lower amounts of another molecule (named “III” in Figure 5a–d), as compared to the GC1237 strain. Genetic complementation of the two mutants with the IE586 cosmid encompassing the region 1653–1697 kb in the genome of *M. tuberculosis* H37Rv, which spans the Rv1503c-6c cluster, restored production of lipids II and III to wild-type levels (Figure 5d). Importantly, genetic complementation of the mutant strains restored significantly their trafficking phenotype, as assessed by LysoTracker staining of infected macrophages (Figure 5e,f). Lipids named “I” to “IV” were purified (Figure 6a for lipid III, Figure 6d for lipid II, not shown for lipids I and IV), and their structure was analyzed by mass spectrometry and NMR. The I to IV lipids were identified as trehalose dimycolates (TDM, data not shown), tetracylated sulfoglycolipid (Ac_4_SGL, Figure 6d–f), 2,3-di-*O*-acyltrehaloses (DAT, Figure 6a–c), and triacylated sulfoglycolipid (Ac_3_SGL, data not shown), respectively. Identification of molecule I as TDM thus explained the very low labelling with [1-^14^C]propionate (Figure 5b,c), as propionate cannot be readily incorporated into this molecule. Lipid quantification using [1-^14^C]acetate did not reveal differences in the amount of TDM produced by the wild-type and the two mutant strains (data not shown). These results show that DAT synthesis is impaired and sulfolipid (mostly Ac_4_SGL) synthesis is increased in the Rv1503c and Rv1506c transposon mutants, as compared to in the wild-type strain. Additional lipids, previously reported to interfere with intracellular trafficking, such as lipoarabinomannan (LAM; [22]) and phosphatidyl-*myo*-inositol mannosides (PIMs; [2]) were analyzed, and we found no difference in the amount and structure of these molecules in the mutant strains, as compared to the wild type strain (Figure S5). These data indicate that deletion of Rv1503c or Rv1506c does not affect the structure and the biosynthesis of *M. tuberculosis* lipoglycans.

**Figure 7:**
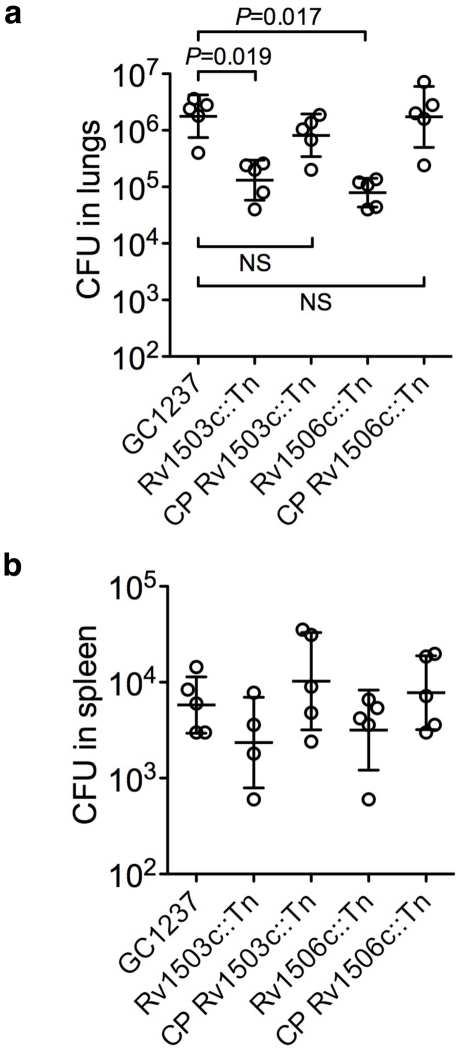
Virulence of the Rv1503c::Tn, Rv1506c::Tn mutant and complemented (CP) strains *in vivo*. Balb/c mice were infected with 10^2^ CFUs of the different strains *via* the intranasal route. After 42 days of infection, mice were sacrificed, and the lungs (a) and spleen (b) homogenized and plated onto agar medium for CFU determination. Each circle represents one animal, and the bars indicate means ± s.d. Differences in the spleen samples were not significant. No differences in the lungs 20 days after infection were observed (not shown).

The two mutants, in particular in Rv1503c (P117C08) grew poorly inside host macrophages (Figure 3c). Interestingly, their ability to colonize the lungs was strongly impaired as assessed in a murine model of *M. tuberculosis* infection, and again the virulence phenotype could be restored by cosmid-mediated complementation (Figure 7).

**Figure 8:**
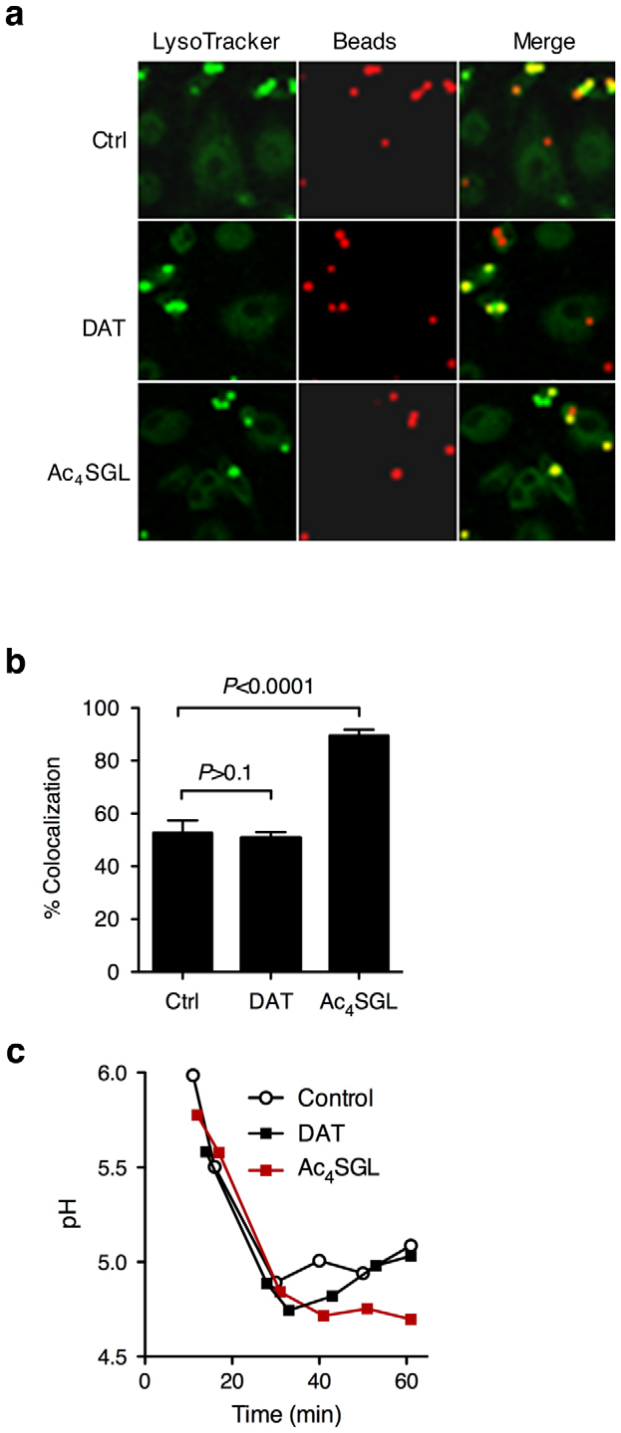
Effect of purified DAT and Ac_4_SGL on intracellular trafficking of silica beads in human macrophages. (**a**) Human monocyte-derived macrophages were pulsed for 15 min with 3-µm silica beads (3-5 beads per cell) coated with BSA-alexa Fluor 647 conjugate (red) and with Ac_4_SGL or DAT. Control beads (ctrl) are not coated with lipids. Cells were chased for 70 min in bead-free medium containing 250 nM LysoTracker Green DND-26 to follow phagosome acidification (green). Cells were fixed and immediately examined under the confocal microscope. (**b**) Phagosome acidification was measured by recording colocalization between the red and the green signals in about 100 phagosomes in 5 independent fields. Data are expressed as mean % of colocalization (+/- s.d.) and are representative of two independent experiments. Data were analyzed using the Student's t-test. (c) Dynamic pH measurement of phagosomes coated with DAT, Ac_4_SGL, and control phagosomes in human macrophages over a 1 h phagocytosis-period. Beads were coated with BSA-FITC and -Alexa 647, and the different lipids, and incubated with human monocyte-derived macrophages for various periods of time (up to one hour). Dual fluorescence was recorded, and the ratio FL_FITC_/FL_647_ was used to calculate the pH values of the phagosomes. A standard curve was constructed after recording the fluorescence values of cells permeabilized and incubated in buffers of defined pH values.

To further dissect the altered trafficking phenotype of the Rv1503c/06c::Tn mutants we evaluated the potential of the DAT and Ac_4_SGL (the major form of sulfolipid overproduced in the mutants) in influencing phagosomal acidification. Using lipid-coated silica beads and LysoTracker staining, we found that phagosomes loaded with Ac_4_SGL-coated beads acidified more than phagosomes containing non-coated beads. In contrast, DAT coating did not influence intracellular trafficking of the particles as compared to non-coated controls (Figure 8a,b). In addition, the pH of the vacuoles containing the different particles was measured using a flow cytometry-based technique [23]. Whereas the pH of the DAT-coated beads and the control beads reached a value of ∼5.2 after 60 min phagocytosis, the pH of the Ac_4_SGL-coated beads was significantly lower (∼4.7) at the same time-point (Figure 8c). These results suggest that overproduction of Ac_4_SGL in the Rv1503c::Tn and Rv1506c::Tn mutant strains may account, at least in part, for their increased intracellular trafficking phenotype.

## Discussion

Here we report the high throughput visual screen of a high density *M. tuberculosis* mutant library for the search of microbial virulence genes involved in phagosome maturation arrest. The power of our approach lies both in: i/the analysis of a single labelling, i.e. “acidic compartments”, to quantify mycobacterial phagosome acidification, which allowed us to exclude any bias due to false co-localizations; ii/the possibility to distinguish between tens of thousands of mutant strains at the single strain level; iii/the possibility of a very stringent selection of the hits.

Many of the genes identified in our study are predicted to be located within operons (*pstS3*, Rv1503c, Rv1506c, *fadD28*, *moaC1*, *moaD1*, Rv3880c) [24]. Using RT-PCR amplification of gene junctions we have confirmed this to be true for the genes Rv1503c-to-Rv1507c on which we focus our detailed analysis (data not shown). Although we could restore the lipid profile in the Rv1506c::Tn mutant by complementing with a plasmid encoding Rv1506c (data not shown), we have chosen as a general strategy to complement our mutants with integrating cosmids. These constructs integrate as single copy into the chromosome and encode the target gene as well as surrounding genes and regulatory elements. This strategy provides for a more realistic reproduction of wild-type gene expression levels of the target gene and has been successfully used in a number of previous studies (e.g. [25]). However, cosmid complementation may complement polar effects of transposon insertion on neighboring genes and does not formally prove the involvement of a single mutated gene in an observed phenotype. The discussion below should be put into this context. The putative involvement of genomic loci is discussed rather than the exact function of given genes.

Here we found only one gene (*fadD28*) that was already shown to play a part in phagosome maturation arrest and has been isolated in a previous similar global approach [10]. In addition, Pethe *et al.* have also identified Rv1522c/*mmpL12*, encoding a putative lipid transporter, as involved in phagosome maturation arrest [10]. Interestingly, this gene belongs to the same genomic locus as the Rv1503c and Rv1506c genes identified here. These three genes may be functionally related. The only modest overlap with datasets from other global screening studies examining related phenotypes [10], [11] may be due to the differences in bacterial strains, host cells and selection techniques employed in the different studies. Indeed, our study is the first to screen for intracellular phenotypes in the GC1237 W-Beijing clinical isolate of *M. tuberculosis* and the selection used was highly stringent, isolating and sequencing only the 10 mutants that gave the strongest LysoTracker signals, *i.e.* 3 s.d. above the mean. It is likely that we have missed some borderline mutants in genes previously reported.

The fatty acid-CoA ligase-encoding gene *fadD28* is required for the synthesis of phtiocerol dimycocerosates (PDIM). These complex lipids have already been shown to be involved in mycobacterial virulence [10], [11], [16], [26], [27], and a recent report has suggested that PDIM may directly prevent phagosome maturation through insertion into the phagosomal membrane [28]. The *lppM* gene was identified as involved in phagosome arrest and encodes a putative lipoprotein that may be anchored in the bacterial membrane. In favour of a membrane modification, the *lppM*::Tn mutant grows in clumps relative to wild type (data not shown). The *ppe54* gene encodes a protein belonging to the large PE/PPE family whose members have mostly been shown to be membrane bound and effective immunogens. Such features have not been reported so far for PPE54. Strikingly, its expression was found specifically upregulated in IFN-γ-activated macrophages [29] suggesting a role in mycobacterial intracellular persistence in agreement with our findings. We also isolated a mutant in Rv3880c, recently named *espL* as part of the ESX-1 secretion system [30]. The exact function of EspL is unknown, however studies in *M. marinum* suggest that proteins encoded by neighboring genes might interfere with phagosome maturation [31]. Other components of the ESX-1 secretome might be involved in phagosome maturation arrest as well [9]. More generally, the ESX-1 secretion system has been involved in mycobacterial virulence in various *in vivo* and *in vitro* systems, including in macrophages [32], [33], [34], [35]. Secreted or membrane-anchored products such as PDIM, lipoproteins, PE/PPE proteins and the ESX-1 secretome might interact with host cell endocytic machinery components yet to be identified.

Additional genes identified here include *pstS3* and genes involved in molybdenum metabolism, namely *moaC1* and *moaD1*. The periplasmic phosphate binding lipoprotein-encoding gene *pstS3* belongs to a larger genetic locus, the *pho* regulon, involved in inorganic phosphate uptake, and previously reported to play a part in mycobacterial virulence [36]. The *pstS3* gene itself has been implicated in mycobacterial intracellular survival, possibly through phagosome maturation arrest [37]. How phosphate uptake is linked to buffering phagosome acidification is unclear and requires further investigation.

The ability of some mutants for intracellular growth despite vacuole acidification is striking and may be explained by several reasons. The exact pH values of the mutants' phagosomes have not been precisely measured here, and it is possible that some mutants reside in more acidic vacuoles than others. As *M. tuberculosis* is highly resistant to very low pH [38], phagosomes of mutants in which acidity is the only altered factor may still be permissive for mycobacterial growth. In addition, as can be seen in Figure 4, a fraction of the phagocytosed mycobacteria does not localize in acidic compartments and thus can be the sub-population that indeed replicates at later time points. It is important to explicitly determine whether the bacterium being observed in a specific sub-cellular compartment is really the one that will replicate, and latest development of life time automated confocal imaging suggest that this may become foreseeable in a near future.

One of the most striking findings of our study is the independent isolation of two pairs of mutants in genes located within the same genetic loci, namely Rv1503c/Rv1506c, and *moaC1*/*moaD1*. Given the size and coverage of our library, it is unlikely that such isolations happened by chance. The adjacent genes *moaC1* and *moaD1* are part of a chromosomal region (Rv3108-20) presumably involved in molybdopterin biosynthesis. We have recently shown that this gene cluster actually forms a genomic island that arose in the ancestor of the tubercle bacilli through horizontal transfer from environmental proteobacteria [39]. Molybdopterin is a precursor of the so-called molybdenum cofactor (MoCo), a coenzyme for various oxidoreductases, including nitrate reductase and sulfite oxidase for instance. Our results are reminiscent of a recent study in which lesion in *moeB1*, another gene possibly involved in MoCo biosynthesis and distant from the Rv3108-20 locus, was shown to affect mycobacterial phagosome biogenesis [9]. The genome of the tubercle bacillus contains several loci potentially involved in MoCo biosynthesis [17], and a few gene products in *M. tuberculosis* harbour molybdopterin/MoCo-binding sites and might use MoCo as a cofactor. This is the case of the nitrate reductase subunit-encoding genes, *narG* and *narX*, of the formate and aldehyde dehydrogenase-encoding genes *fdhF* and *nuoG*, respectively, as well as of two other gene products, Rv0197, an uncharacterized oxidoreductase, and Rv0218, a possible sulfite oxidase. Although we can only speculate at this stage, we can anticipate that MoCo-dependent redox reactions play a crucial part in early intracellular fate of the tubercle bacillus, which should deserve further attention in future studies.

We also isolated two independent mutants carrying genetic disruptions in Rv1503c and Rv1506c. The homologues of these genes in *M. marinum* belong to a locus involved in the synthesis of LOS [13]. Here we showed that, like other *M. tuberculosis* strains [19], [20], our W-Beijing mother strain does not synthesize LOS. However we showed that the Rv1503c::Tn and Rv1506c::Tn mutants are impaired in the synthesis of other acyltrehalose-containing lipids, namely DAT, and that they overproduce sulfoglycolipids (SGL), Ac_3_SGL and more importantly Ac_4_SGL. DAT and SGL are glycolipids based on trehaloses [40]. Although some of the genes involved in DAT [40], [41] and SGL [42], [43], [44] synthesis have been identified, knowledge of the enzymes, transporter and regulators involved in these pathways is still incomplete. Transcriptional regulation of the genes involved in synthesis of these molecules has been shown to be coordinated by the PhoP regulator [15]. This suggests that Ac_4_SGL/Ac_3_SGL overproduction in the mutant strains may be due to a compensation mechanism associated with impairment of DAT production. However, the Rv1503c and Rv1506c proteins do not carry the signature of enzymes such as acyltransferases and polyketide synthases (Pks), and it is thus unclear what their exact involvement in DAT synthesis could be. The Rv1505c gene encodes a putative acyltransferase and the Rv1505c and Rv1506c genes are predicted to form an operon; the possibility thus remains that impairment of DAT synthesis in the Rv1506c mutants, and eventually in the Rv1503c mutants, is actually due to inactivation of Rv1505c because of operon disruption and/or of polar effects. Interestingly, transcription of Rv1505c, as well as that of other genes located in the same genomic region, namely Rv1517, Rv1518, Rv1522c/*mmpL12*, Rv1525/*wbbL2*, Rv1527c/*pks5* and Rv1528c/*papA4*, has been proposed to be regulated by PhoP [45]. Whether and how these genes are involved in DAT synthesis will require further investigation and is beyond the scope of the present study.

Most importantly, our results suggest that Ac_4_SGL, but not DAT, increase phagosome acidification. This is at variance with conclusions from an early study suggesting that mycobacterial sulfolipids (SL) tend to impair phagosome maturation [46]. However these authors incubated purified sulfolipids directly with the cells, and the model systems used in this study and in ours are barely comparable. Nevertheless, our results obtained with beads suggest that SL increase phagosome maturation, which may account, at least in part, for the increased trafficking of the Rv1503c and Rv1506c mutants, although the exact mechanism of increased acidification of these two mutants has not been formally identified here. Furthermore, analysis of the production of cytokines and chemokines using protein arrays upon infection with Rv1503c and Rv1506c mutants did not reveal any significant differences compared to the wild type strain (data not shown). This suggests that the mutants altered trafficking phenotypes were not caused by changes in their ability to induce different inflammatory cytokine production [47], [48] and indicates that other mechanisms might account for the observed trafficking behaviour. For instance, other possible explanations include an overall altered cell wall, or a more generally reduced intracellular fitness of the mutants, which both may impact intracellular trafficking. In this regard, it is interesting to notice that the *phoP* mutant shows a significant, though partial, relocation to an acidic phagosome (Figure 1). The PhoP transcriptional regulator controls the expression of many genes, some of which have been involved in phagosome remodelling; this is the case of *fadD28*, *fadD26* and *mmpL12* for instance [10], [45]. It also regulates the synthesis of important lipids, such as diacyltrehaloses and sulfolipids [15], which may be involved in intracellular trafficking according to our data. The partial inability of the *phoP* mutant to arrest phagosome maturation may thus be due to a defect in production of these factors. It may also be due to a broader defect in fitness [49]. Further study of our mutants and other mutants isolated in previous screens will help understand the general relationship that may exist between mycobacterial fitness and intracellular trafficking.

In summary, the unbiased approach developed here allowed us to identify novel mycobacterial genes involved in *M. tuberculosis* intracellular parasitism, and suggests the involvement of important *M. tuberculosis*-specific glycolipids in this process. This approach can easily be adapted for the comprehensive quantitative analysis of mycobacterial vacuole sorting, as well as for the study of intracellular parasitism by pathogenic microorganisms, and for phenotypic drug screening [50].

## Methods

### Bacteria and mutant library

*M. tuberculosis* H37Rv, GC1237[14] and GC1237 Δ*phoP/R* [15] strains were grown in Middlebrook 7H9 culture medium (Difco, Sparks MD) supplemented with 10% oleic acid-albumin-dextrose-catalase (OADC, Difco), glycerol, 0.05% Tween 80, and 25 µg/ml kanamycin in the case of the Δ*phoP/R* mutant. A library of 11,180 members was constructed using *M. tuberculosis* Beijing GC1237 as a host strain and the pCG113 plasmid that contains the IS*1096*-derived Tn5367 as previously described [16]. After electroporation, transformants were amplified in Middlebrook 7H9 culture medium supplemented with 10% OADC, glycerol, 0.05% Tween 80 and 25 µg/ml kanamycin at 32°C, a temperature that allows replication of circular pCG113. The mutants were selected on Middlebrook 7H10-OADC agar medium supplemented with 25 µg/ml kanamycin and 2% sucrose at 39°C. This temperature, and the presence of sucrose, allows to select mutants in which the plasmid has been eliminated and the double cross over has occurred. Eleven thousand one hundred and eighty individual mutant clones were isolated and seeded into 96-well plates containing 180 µl of Middlebrook 7H9 culture medium supplemented with 10% OADC, glycerol, 0.05% Tween 80 and 25 µg/ml kanamycin. The plates were incubated at 37°C for three weeks and then kept at 4°C. Three weeks before screening, a 10-fold dilution of suspension from each of the *M. tuberculosis* transposon mutants was performed. Selected strains were transformed with a GFP-expressing integrative plasmid kindgly provided by Dr. Winter (Institut Pasteur, Paris). Transformants were selected on hygromycin.

### TraSH analysis

Mapping of transposon insertion sites by microarray was carried out essentially as described previously [11]. Genomic DNA was prepared from a pool of 500 Tn*5367* transposon mutants and digested with BssH*II* and Mlu*I*. Approximately 100 ng of digested genomic DNA was ligated to 100 pmol of Y-linker [11], and then 10 ng of ligated DNA was used as template for PCR amplification of transposon-flanking regions using the transposon-specific primer IS1 (5′-GCACGTCGAGGTCTTTCAGATGGATGGCG-3′) and the Y-linker-specific primer TA4 (5′-ACGCACGCGACGAGACGTAGC-3′) in the presence of 8% DMSO. Following an initial denaturation step for 2 minutes at 95°C, the reaction was hot-started and cycled between 94.5°C (30 s) and 72°C (90 s) for 22 cycles. Amplification products were gel purified and used in a second round of PCR amplification between the Y-linker-specific primer TA4 and a nested transposon-specific primer IS2Nest (3′-TGGATGGCGTAGGAACCTCCATCATCGGA-5′) to further enrich for transposon-flanking products and to incorporate Cy3-dCTP (Amersham). The reaction mix included 20 µM Cy3-dCTP, 180 µM dCTP and 200 µM dGTP/dATP/dTTP and the cycling conditions were as before, but for 14 cycles. The labelled PCR products were cleaned up using a Qiagen MinElute kit, eluting in water.

The fluorescently labelled transposon insertion sites were hybridised to whole genome microarrays, prepared by spotting the *M. tuberculosis* 70-mer oligonucleotide set (Operon, Qiagen) onto Corning GAPS Coated Slides. Signal intensities of hybridisation were collected using Genepix Pro 3.0 and an Axon 4000B microarray scanner.

### Controls preparation and mycobacteria staining

Bacteria were harvested, washed three times and resuspended in phosphate buffer saline (PBS). The bacteria were then sonicated and allowed to stand for 30 minutes to allow residual aggregates to settle. The bacterial suspensions were then aliquoted and frozen at −80°C. A single defrosted aliquot was used to quantify the CFUs prior to inoculation and typical stock concentrations ranged between 2 and 5×10^8^ CFUs/ml. Dead bacteria were prepared by heating one aliquot at 95°C for 20 minutes. Where appropriate, mycobacteria were covalently labeled with CypHer5 mono ester dye (Sigma Aldrich, Saint-Louis, MO) in 0.1 M sodium carbonate buffer (pH 9) and washed three times before use [51]. Three µm size-Zymosan A from *Saccharomyces cerevisiae* (Sigma Aldrich) was titrated at 1.5×10^6^ particules/ml.

### Macrophages, infection, and fluorescence assay set up

Mouse bone-marrow-derived macrophages were obtained by seeding 10^7^ bone marrow cells from C57BL/6 mice in 75 cm^2^ dishes in RPMI 1640 supplemented with 10% heat-inactivated fetal calf serum (FCS) and 10% L-cell conditioned medium (all from Gibco at Invitrogen, Carlsbad, CA). Peripheral Blood Mononuclear Cells (PBMC) were isolated from buffy coat from healthy volunteers. 15 ml of Ficoll-Paque Plus (Amersham Biosciences, Sweden) were added to PBS diluted buffy coat diluted and centrifuged at 2500×*g* for 20 min. PBMC were obtained by CD14^+^ beads separation (Miltenyi Biotec, Germany), washed 3-times with PBS containing 1% FCS and transferred to 75 cm^2^ culture flask containing RPMI 1640 media, 10% FCS and 50 ng/ml of recombinant-human macrophage colony stimulating factor (rh-MCSF, R & D systems, Minneapolis). After 6 days, murine or human macrophages were harvested with Versene (Gibco) and seeded at a density of 1.5×10^5^ cells per well in 384-well plates (Evotec, Hambourg, Germany) in 50 µl RPMI 1640 supplemented with 10% heat-inactivated fetal calf serum and 10% L-cell conditioned medium. Adherent cells were then infected with bacterial suspensions at a MOI varying from 20 to 1 bacteria per cell and incubated for 2 h. Cells were then washed three times with PBS supplemented with 1% FCS and further incubated with 2 µM LysoTracker green DND-26 (Invitrogen) for 1.5 h. Finally, cells were fixed with 1.5% formaldehyde BD Lyse solution (BD Biosciences, San Jose, CA) for 10 min, washed twice and stained with 5 µg/ml DAPI dilactate (Sigma) in 0.1% Triton X-100 (Sigma) in PBS. In some preparations, fixed cells were labelled with an anti-Rab5A (Abcam, Ab13253, 1/500 dilution), -LAMP-1 (Cell Signaling, C54H11, 1/200), -v-ATPase (Synaptic Systems, 109002, 1/100 dilution), and -CD63 (Caltag Laboratories, 18-7300, dilution 1/100) antibodies, subsequently detected using a FITC-, Alexa 555-, or rhodamine-conjugated rabbit anti-mouse IgG or mouse anti-rabbit antibody (Sigma or Invitrogen).

### Image acquisition by automated confocal microscopy and data analysis for Lysotracker assay- set-up

Confocal images were recorded on an automated fluorescent confocal microscope Opera (Evotec) using a 20X water objective (NA 0.70), 405-nm, 488-nm and 635-nm lasers and a 510 primary dichroic mirror with two sequential exposures. Each image was then processed using dedicated in-house image analysis software (IM). Each recorded field contained three images (three colors): one for the cell nuclei (blue-channel), one for the LysoTracker-DND-26 positive compartment (green-channel) and one for the CypHer5 labeled mycobacterium-positive compartment (red-channel). Cell nuclei, mycobacteria and LysoTracker-DND-26 positive compartment are then segmented in the “nuclei” band, “bacteria” and the “acidic compartment” band respectively using in-house method [52]. Briefly, this method relies on a succession of i) thresholding the histogram of the original image, the threshold being taken as the maximum value between a manual threshold and an automatic one (lowest value of a 3 classes K-means); ii) Gaussian filtering the original image with a standard deviation that is set equal to the nuclei (/mycobacterium and acidic compartments) average radius; iii) searching for local maxima of the filtered image that provides nuclei (/mycobacterium and acidic compartments) centers as seeds for iv) region growing that defines the individual surface of each nucleus (/mycobacterium and acidic compartments) and finally v) removing extremely small nuclei (/mycobacterium and acidic compartments) as potential artifacts or noise. Once mycobacterium and acidic compartments and nuclei are individually identified, final results are i) the nuclei number, ii) the number of nuclei that are proximal to at least one mycobacterium or acidic object, iii) the surface covered by acidic or mycobacterium compartments in the vicinity of the nuclei. Proximity is a parameter manually set-up by the user. Given the fact that one cell has only one nucleus, these results can be extrapolated to i) the number of cells, ii) the number of cells containing at least one acidic object/one mycobacterium, and iii) the surface of acidic compartments or bacterial load proximal to cell nuclei. Final results are expressed as the average over all the images of the well.

### Mutant library screening

Frozen bone-marrow progenitors (6×10^8^ cells) were seeded in three 500 cm^2^ dishes in RPMI 1640 supplemented with 10% FCS and 10% L-cell conditioned medium. At day 5, 50 µl of *M. tuberculosis* Beijing GC1237 mutants were two-fold diluted in PBS and seeded into a 96-well plate (Nunc), and 20 µl of the diluted suspension were subsequently transferred into a 384-well assay plate using the BioTek Precision XS sample processor. Before infecting cells, the concentration of each mutant was determined by OD_600_ measurement and the mean titer was within the range of 0.2 to 0.6 showing that the mycobacteria were in exponential growth phase (data not shown). Plate titration was performed on randomly selected mutants and the mean titer was 2×10^7^ colony forming units (CFUs)/ml. 20 µl of heat-killed *M. tuberculosis* Beijing GC1237 Δ*phoP/R*, *M. tuberculosis* Beijing GC1237 Δ*phoP/R* and wild type strains were added manually in column 2, 3 and 22 respectively. The next day, after harvesting, 30 µl of the cell suspension at 5×10^5^ cells/ml was distributed into the 384-well assay plate that had been priory seeded with the mutants using the AquaMax DW4 liquid handling device (Molecular Devices). After a 2 h incubation at 37°C under 5% CO_2_, extracellular mycobacteria were removed by 3 washes in PBS and macrophages were labeled with 2 µM LysoTracker red DND-99 (Invitrogen) for 1.5 h. Finally, cells were fixed with 1.5% formaldehyde BD Lyse solution for 10 min, stained with 5 µg/ml DAPI in 0.1% Triton X-100 (Sigma) in PBS for 2 min and kept in 1% FCS in PBS at 4°C until image acquisition.

### Image acquisition by automated confocal microscopy and data analysis for high throughput screening (HTS)

Confocal images were recorded on an automated fluorescent confocal microscope Opera (Evotec) using a 20X water objective (NA 0.70), 405-nm and 561-nm lasers and a 580 primary dichroic mirror. Each image was then processed using dedicated in-house image analysis software (IM). Images contained two bands (two colors): one for the LysoTracker-positive compartment (red-channel) and one for the cell nuclei (blue-channel). The image dynamics in both bands (both colors) was first checked through a series of statistical tests including average intensity and standard deviation designed to remove black or out of focus images. Cell nuclei and acidic compartments are then segmented in the “nuclei” band, and the “acidic compartment” band respectively using in-house method [52]. Briefly, this method relies on a succession of i) thresholding the histogram of the original image, the threshold being taken as the maximum value between a manual threshold and an automatic one (lowest value of a 3 classes K-means); ii) Gaussian filtering the original image with a standard deviation that is set equal to the nuclei (/acidic compartments) average radius; iii) searching for local maxima of the filtered image that provides nuclei (/acidic compartments) centers as seeds for iv) region growing that defines the individual surface of each nucleus (/acidic compartments) and finally v) removing extremely small nuclei (/acidic compartments) as potential artifacts or noise. Once acidic compartments and nuclei are individually identified, final results are i) the nuclei number ii) the number of nuclei that are proximal to at least one acidic object iii) the surface covered by acidic compartments in the vicinity of the nuclei. Proximity is a parameter manually set-up by the user. Given the fact that one cell has only one nucleus, these results can be extrapolated to i) the number of cells) ii) the number of cells containing at least one acidic object and iii) the surface of acidic compartments proximal to cell nuclei. Final results are expressed as the average over four fields recorded per 384-plate well.

### Statistical analysis

The screen statistical data (Z', CV etc. for the control plates) was calculated using an in-house software. Additional analyses with regards to quality control were performed with the Spotfire software.

### Genetic analysis of the mutants and complementation

The transposon insertion sites were identified by ligation mediated PCR following a slightly modified version of a previously described protocol [53]. Briefly, genomic DNA was digested with BamH*1*, Xho*I* or Bgl*II*. DNA was then ligated to BamH*I*-linkers, and amplified as described. The Rv1503c and Rv1506c mutants were complemented as previously described [27], with the pYUB412-derived cosmid [18] MTCI586, which carries a 38.7- and 20.8-kb DNA fragment covering the 1653- to 1697-kb region of the *M. tuberculosis* chromosome. This cosmid encompasses the Rv1503c-6c gene cluster (1694.5 to 1696.4 kb, genomic coordinates). A description of the other cosmids used in the study is provided in Table 2.

**Table 2:** List of the cosmids used to complement the mutants.

Mutant id	Gene (Rv n°)	Cosmid number Region spanning relative to Rv (kb)	Genes covered
P69D07	*pstS3* (Rv0928)	I362 (1029-1071)	Rv0923c-0957
P117C08	Rv1503c (Rv1503c)	IE586 (1653-1697)	Rv1446-506c
P2E07	Rv1506c (Rv1506c)	IE586 (1653-1697)	Rv1446-506c
P65B12	*lppM* (Rv2171)	I135 (2409-2442)	Rv2151c-78
P1E07	Rv2295 (Rv2295)	I238 (2563-2599)	Rv2264c-98
P58C04	*fadD28* (Rv2941)	I49 (3258-3292)	Rv2934-45c
P32E07	*moaC1* (Rv3111)	I528 (3454-3485)	Rv3088-117
P36D07	*moaD1* (Rv3112)	I528 (3454-3485)	Rv3088-117
P55C04	*ppe54* (Rv3343c)	I275(3729-3761)	Rv3343c-9c
P39E07	Rv3880c (Rv3880c)	RD1-2F9 (4337-4369)	Rv3861-85c

### Preparation, purification and analysis of lipids

Lipids were extracted from 21-day old *M. tuberculosis* cultures first by adding 2 volumes of CH_3_OH and 1 volume of CHCl_3_ for 2 days, then in 2∶1 CHCl_3_/CH_3_OH (v/v). Pooled extracts were concentrated, washed with water and evaporated to dryness. For specific radiolabeling, each strain was cultured to exponential phase and labeled by incubation with [1-^14^C]propionate (54 Ci.mol^−1^, ARC Radiochemical, St Louis, MO) for 2 days. Lipids were first analyzed on silica gel 60 TLC plates (E. Merck, Darmstadt, Germany) in various solvent systems (see the legend to figures). Radiolabeled lipids were visualized with a Typhoon PhosphorImager (Amersham, Biosciences). For lipid purification, the chloroform phase was applied to an anion exchange Sep-pak cartridge (Waters Accell Plus QMA; Waters Corporation) eluted successively by 10 ml CHCl_3_, 10 ml of CHCl_3_/CH_3_OH 95/5 (v/v), 10 ml of CHCl_3_/CH_3_OH 9/1 (v/v) to elute DAT, 10 ml of CHCl_3_/CH_3_OH/H_2_O 60/35/8 (v/v) to elute residual neutral compounds, 10 ml of chloroform/methanol 1/2 (v/v) containing 0.1 M ammonium acetate to elute negatively charged compounds (phospholipids) and 10 ml of chloroform/methanol 1/2 (v/v) containing 0.3 M ammonium acetate for the elution of sulfolipids. PIMs were extracted from bacterial cells and subjected to MALDI-MS analysis in the negative ion mode as described previously [54].

### MALDI-*Tof*-MS

MALDI-*Tof*-MS analysis were performed on a 4700 Proteomics Analyser (with Tof-Tof Optics, Applied Biosystems) using the reflectron mode. Ionization was effected by irradiation with pulsed UV light (355 nm) from an Nd:YAG laser. Samples were analyzed by the instrument operating at 20 kV in the positive ion mode using an extraction delay time set at 20 ns. Typically, spectra from 1,000 to 2,500 laser shots were summed to obtain the final spectrum. The HABA (2-[4-hydroxy-phenylazo]-benzoic acid) matrix was used at a concentration of ∼10 mg/ml in ethanol/water (1:1, v/v). Then, 0.5 µl sample solution and 0.5 µl of the matrix solution were deposited on the target, mixed with a micropipette and dried under a gentle stream of warm air. The measurements were externally calibrated at two points with mycobacterial PIM.

### Chemical analysis of the mannose cap on LAM

The presence of mannose caps in *M. tuberculosis* strains GC1237, Rv1503c::Tn and Rv1506c::Tn was analyzed by capillary electrophoresis (CE) as described earlier [55]. In short, partially purified ManLAM is partially degradated by controlled acid hydolysis (0.1 M HCl for 20 min. at 110°C), and the oligosaccharides liberated tagged with the fluorescent label 8-aminopyrene-1,3,6-trisulfonate (APTS). During CE, the labeled oligosaccharides are separated and peaks are detected by laser-induced fluorescence and elution times compared with the appropriate standards.

### NMR analysis

NMR spectra were recorded with an Avance DMX500 spectrometer (Bruker GmbH, Karlsruhe, Germany) equipped with an Origin 200 SGI using Xwinnmr 2.6. Native molecules were dissolved in CDCl_3_-CD_3_OD, 9:1, v/v and analyzed in 200×5 mm 535-PP NMR tubes at 295 K. Proton chemical shifts are expressed in ppm downfield from the signal of the chloroform (d_H_/TMS 7.27). All the details concerning used COSY, HOHAHA and ^1^H-^13^C HMQC sequences and experimental procedures were as previously reported [56].

### Trafficking study with silica beads

Silica beads (3 µM diameter; Kisker Biotech, Germany) were washed two times with sterile PBS before incubation overnight at 4°C with 5% (w/v) BSA-Alexa Fluor 647 (Invitrogen, Eugene, OR). The beads were then washed two times with PBS to remove unbound BSA. BSA coated beads were added in a glass tube containing 100 µg of DAT or Ac_4_SGL and sonicated for 10 minutes. All coated beads were washed once again two times with PBS before trafficking experiments. The efficiency of lipid coating was evaluated by re-extracting the lipids from the coated beads followed by TLC analysis, and was found to be nearly 100% (not shown).

Lipid-coated beads suspended in RPMI (+10% human serum) were added at a ratio of 3-5 beads/cell on human macrophages differentiated from adherent mononuclear cells from healthy donor in the presence of 5 UI/ml of hMCSF (Miltenyi Biotech, Germany) for 6 days in 48-well plates. Plates were centrifuged for 1 minute at 1,000 rpm and incubated at 37°C under 5% CO2. Fifteen minutes later, cells were washed with PBS and 200 µl RPMI containing 250 nM of LysoTracker green DND-26 (Invitrogen) was added before incubation at 37°C under 5%CO_2_. Cells were fixed 70 minutes later with 4% PFA and the number of LysoTracker-positive phagosomes was immediately estimated using a Leica confocal fluorescence microscope (SP2) equipped with a Plan Apo 4061.4 Ph 6 objective (Olympus Optical) and CoolSNAP-Pro CF digital camera in conjunction with Image-Pro Plus version 4.5.1.3 software (Media Cybernetics). At least 500 beads from at least 5 independent fields were counted for each experiment. For phagosomal pH quantification, a flow cytometry-based method of dual fluorescence measurement was employed [23]. Briefly, beads were coated with 5% FITC- and Alexa Fluor 647-conjugated BSA in PBS overnight at 4°C. Beads were washed twice in PBS, and sonicated in PBS in the presence of the different purified lipids (100 µg lipids/2.10^6^ beads) at room temperature for 15 min. Beads were washed twice in PBS, and used to pulse the cells (2 beads/cell). Fluorescence recording (LSR II apparatus, Becton Dickinson, San Jose, CA) and pH calculation were done as previously and extensively detailed in [23].

### *In vivo* experiments

6 week-old female Balb/C mice (ORIENTBIO Inc., South Korea) were challenged with *M. tuberculosis* GC1237, Rv1503c::Tn, Rv1506c::Tn and their corresponding complemented counterparts *via* the intranasal route with 10 µl of a suspension containing 5.10^5^ organisms/ml to obtain an inhaled dose of 100 CFU in lungs. After 42 days, organs from killed mice were homogenized by use of an MM300 apparatus (Qiagen) and 2.5-mm diameter glass beads. Serial 10-fold dilutions in medium were plated on 7H11 agar and colony forming unit counts were ascertained at 37°C after 3 weeks of growth.

### Ethics statement

Animal studies were carried out in strict accordance with the recommendations from the Animal Protection Law in Korea. The protocol was approved by the Institutional Animal Care and Use Committee of Institut Pasteur Korea (Permit Number: IPK-10003). All efforts were made to minimize suffering of the animals. Human monocytes were purified from blood samples obtained from healthy blood donors under strict anonymity (Etablissement Français du Sang, EFS, Toulouse). Written informed consents were obtained from the donors under EFS contract n°21/PVNT/TOU/IPBS01/2009-0052. With respect to Decree n°2007-1220 (articles L1243-4, R1243-61 and following) dated August 10th 2007 of the French Public Health Code (published in the Official Journal of the French Republic of August 14th 2007), the contract received formal approval by the French Ministry of Science and Technology (decision n°AC 2009-921). As a “collection” is defined by Decree 2007-1220 as “a collection, for scientific use, of biological samples from a group of persons who have been identified and selected on the basis of clinical or biological characteristics of one or several members of the group”, and as the blood samples used in our study were not obtained from individuals that have been “identified or selected on the basis of clinical or biological characteristics”, these samples do not constitute a so-called “collection”, which waives the need for ethical approval by the ethical committee “Comité de Protection des Personnes”, in agreement with Decree 2007-1220, article R1243-63.

## Supporting Information

Figure S1 *M. tuberculosis* and Zymozan intracellular localization in macrophages as determined by automated confocal microscopy. (**a**) Representative pictures of mouse bone marrow-derived macrophages infected with live or heat-killed (HK) DsRed-expressing *M. tuberculosis* GC1237. After 2 hours of infection, cells were stained in blue with DAPI (nuclei), and in green with LysoTracker (acidic compartments), or with a FITC-conjugated anti-Rab5 (early endosomes) antibody. Infected cells stained with a FITC-conjugated isotype antibody are shown as control. Images span 0.450×0.340 mm^2^. (**b**) Cells were incubated for 2 hours with red Zymozan, and stained with LysoTracker (green). Images span 0.450×0.340 mm^2^. (**c**) Quantification of zymozan-LysoTracker co-localization in function of Zymozan amount.
(0.62 MB TIF)

Figure S2 Insertion distribution of Tn5367 in the *M. tuberculosis* GC1237 mutant library. Insertion sites in a pool of 500 mutants were detected by fluorescent labelling of transposon-flanking DNA and hybridisation to a whole-genome microarray.
(0.40 MB TIF)

Figure S3 Detection of CD63 (A) and V-ATPase (B) in phagosomes containing the wild type and Rv1503c and Rv1506c mutant strains. Human monocyte-derived macrophages were infected with FITC-labelled bacteria at an MOI of 10 for 1 h, after which cells were washed and further incubated in fresh medium for another 2 h. Cells were fixed, the markers were immuno-detected (red signal), and cells were observed under the confocal microscope. The histograms in the right panels show co-localization quantification after counting 100-150 phagosomes in about 10 fields. Data are expressed as mean % of colocalization (+/− s.d.) and are representative of two independent experiments. Data were analyzed using the Student's t-test. ***, *P*<0.001.
(1.36 MB TIF)

Figure S4 Growth of the selected attenuated mutants and the GC1237 wild type strain in 7H9-ADC broth.
(0.20 MB TIF)

Figure S5 Lipoglycan analysis in *M. tuberculosis* wild type and Rv1503c and Rv1506c mutant strains. (a) MALDI-MS spectra of phosphatidyl-myo-inositol mannosides (PIM) composition of *M. tuberculosis* GC1237 and the Rv1503c::Tn and Rv1506c::Tn mutants. (b) Mannooligosaccharide cap analysis of ManLAM by capillary electrophoresis (CE). Partially purified ManLAM from *M. tuberculosis* Beijing GC1237 (trace 1), and the Rv1503c::Tn and Rv1506c::Tn mutants (traces 2 and 3, respectively) is analysed for the presence of the mannose caps by CE. Peak I: APTS; II: Ara-APTS; III: Man-APTS; IV: internal standard; VI: Man*p*-Man*p*-Ara-APTS; VII: Man*p*-Man*p*-Man*p*-Ara-APTS.
(0.30 MB TIF)
