# Supplementary figures and images for: High Content Phenotypic Cell-Based Visual Screen Identifies Mycobacterium tuberculosis Acyltrehalose-Containing Glycolipids Involved in Phagosome Remodeling
Source: PLoS Pathog. 2010 Sep 9;6(9):e1001100. doi: 10.1371/journal.ppat.1001100 (PMC2936551; doi:10.1371/journal.ppat.1001100)

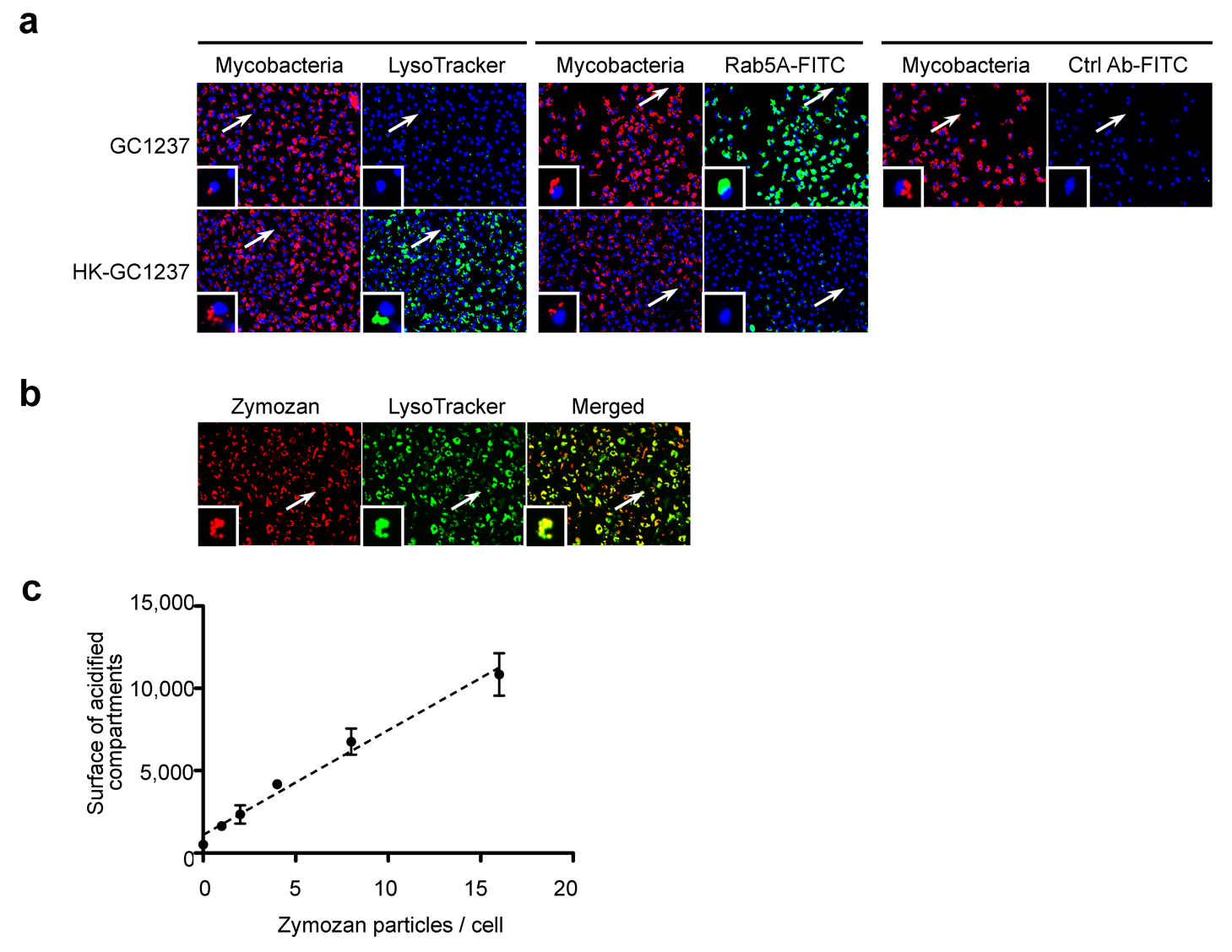

Supplement: Figure S1 — M. tuberculosis and Zymozan intracellular localization in macrophages as determined by automated confocal microscopy. (a) Representative pictures of mouse bone marrow-derived macrophages infected with live or heat-killed (HK) DsRed-expressing M. tuberculosis GC1237. After 2 hours of infection, cells were stained in blue with DAPI (nuclei), and in green with LysoTracker (acidic compartments), or with a FITC-conjugated anti-Rab5 (early endosomes) antibody. Infected cells stained with a FITC-conjugated isotype antibody are shown as control. Images span 0.450×0.340 mm2. (b) Cells were incubated for 2 hours with red Zymozan, and stained with LysoTracker (green). Images span 0.450×0.340 mm2. (c) Quantification of zymozan-LysoTracker co-localization in function of Zymozan amount. (0.62 MB TIF) [file ppat.1001100.s001.tif]

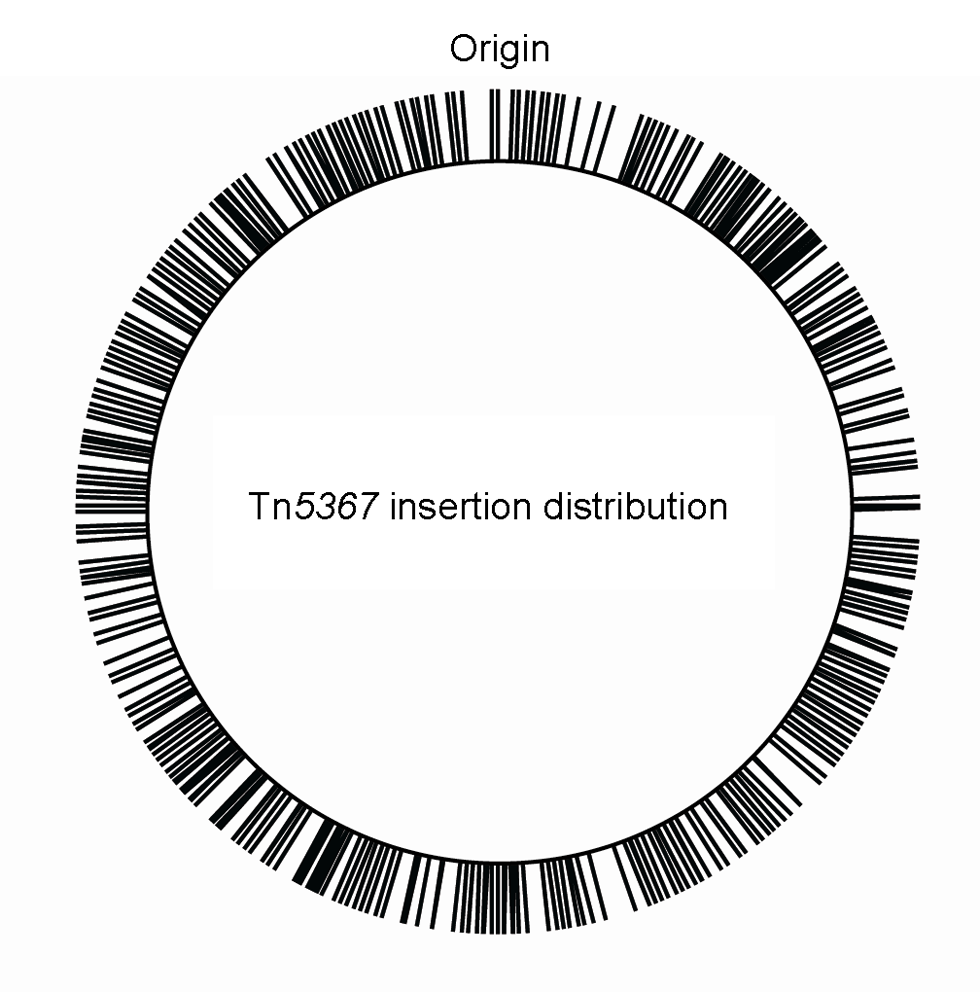

Supplement: Figure S2 — Insertion distribution of Tn5367 in the M. tuberculosis GC1237 mutant library. Insertion sites in a pool of 500 mutants were detected by fluorescent labelling of transposon-flanking DNA and hybridisation to a whole-genome microarray. (0.40 MB TIF) [file ppat.1001100.s002.tif]

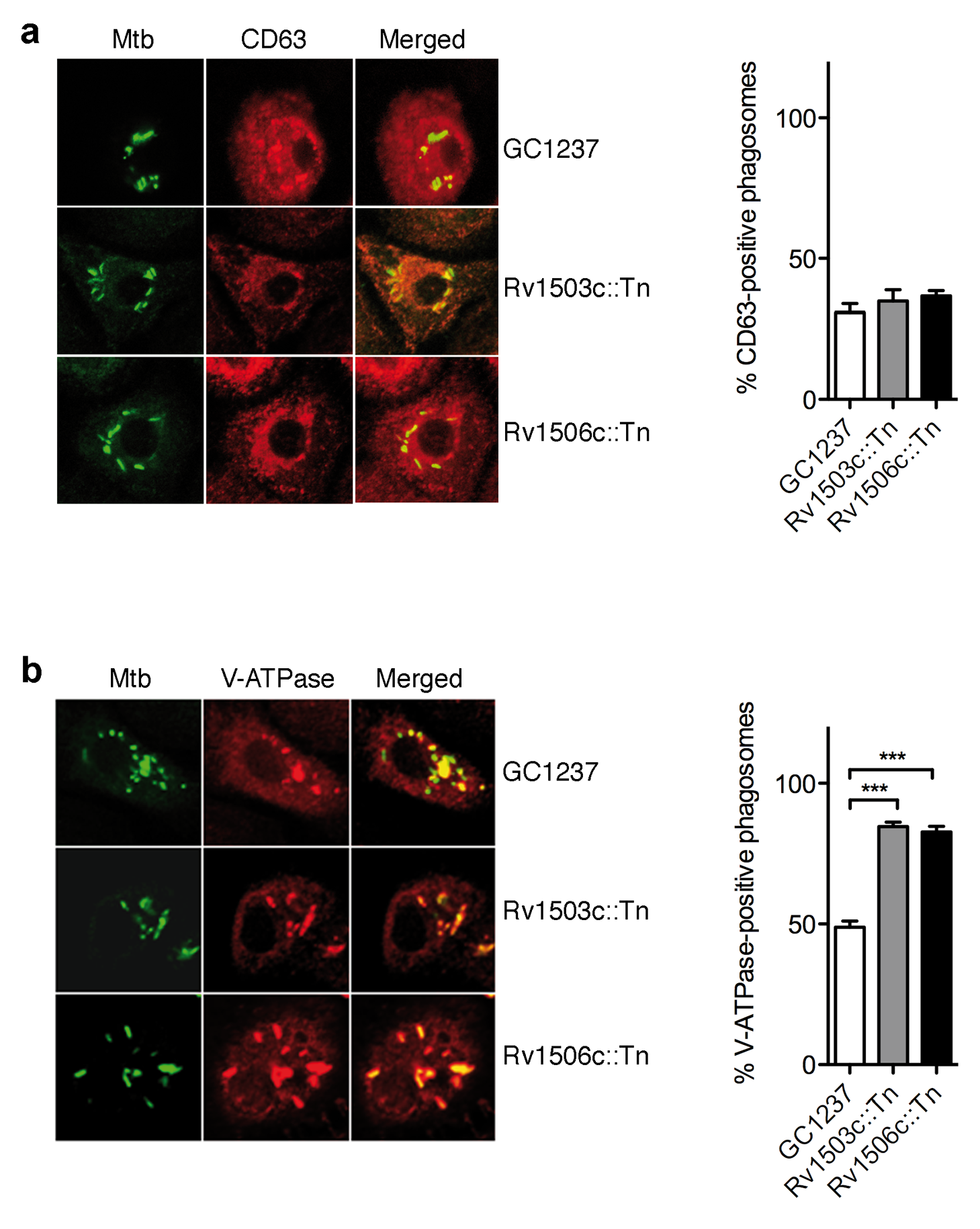

Supplement: Figure S3 — Detection of CD63 (A) and V-ATPase (B) in phagosomes containing the wild type and Rv1503c and Rv1506c mutant strains. Human monocyte-derived macrophages were infected with FITC-labelled bacteria at an MOI of 10 for 1 h, after which cells were washed and further incubated in fresh medium for another 2 h. Cells were fixed, the markers were immuno-detected (red signal), and cells were observed under the confocal microscope. The histograms in the right panels show co-localization quantification after counting 100-150 phagosomes in about 10 fields. Data are expressed as mean % of colocalization (+/− s.d.) and are representative of two independent experiments. Data were analyzed using the Student's t-test. ***, P<0.001. (1.36 MB TIF) [file ppat.1001100.s003.tif]

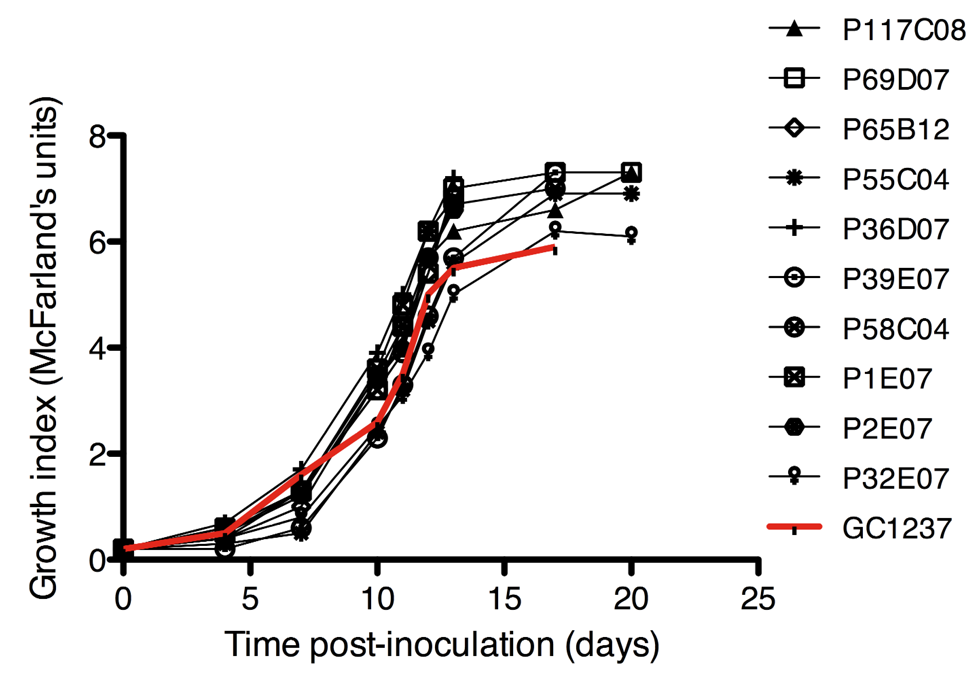

Supplement: Figure S4 — Growth of the selected attenuated mutants and the GC1237 wild type strain in 7H9-ADC broth. (0.20 MB TIF) [file ppat.1001100.s004.tif]

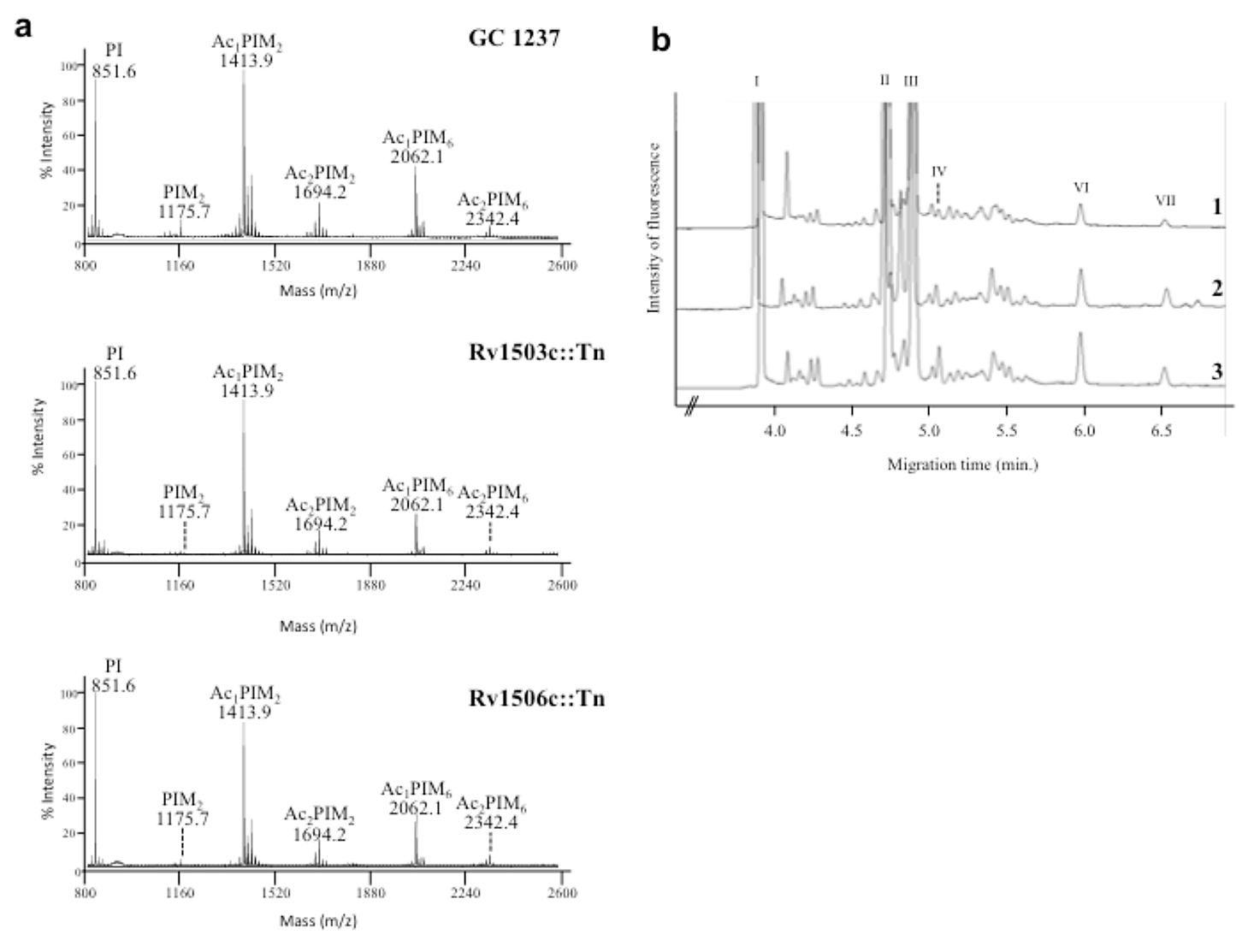

Supplement: Figure S5 — Lipoglycan analysis in M. tuberculosis wild type and Rv1503c and Rv1506c mutant strains. (a) MALDI-MS spectra of phosphatidyl-myo-inositol mannosides (PIM) composition of M. tuberculosis GC1237 and the Rv1503c::Tn and Rv1506c::Tn mutants. (b) Mannooligosaccharide cap analysis of ManLAM by capillary electrophoresis (CE). Partially purified ManLAM from M. tuberculosis Beijing GC1237 (trace 1), and the Rv1503c::Tn and Rv1506c::Tn mutants (traces 2 and 3, respectively) is analysed for the presence of the mannose caps by CE. Peak I: APTS; II: Ara-APTS; III: Man-APTS; IV: internal standard; VI: Manp-Manp-Ara-APTS; VII: Manp-Manp-Manp-Ara-APTS. (0.30 MB TIF) [file ppat.1001100.s005.tif]
